# High-throughput *in situ* perturbation of metabolite levels in the tumor micro-environment reveals favorable metabolic condition for increased fitness of infiltrated T-cells

**DOI:** 10.3389/fcell.2022.1032360

**Published:** 2022-12-22

**Authors:** Veronica Valvo, Elena Parietti, Kyle Deans, Sebastian W. Ahn, Noel Ruth Park, Benjamin Ferland, Devon Thompson, Christine Dominas, Sharath K. Bhagavatula, Shawn Davidson, Oliver Jonas

**Affiliations:** ^1^ Department of Radiology, Brigham and Women’s Hospital, Harvard Medical School, Boston, MA, United States; ^2^ Department of Infectious Diseases and Hospital of Epidemiology, University Hospital of Zurich, University of Zurich, Zurich, Switzerland; ^3^ Lewis-Sigler Institute for Integrative Genomics, Princeton University, Princeton, NJ, United States

**Keywords:** tumor micro-environment, immunometabolism, cancer metabolism, immunotherapy, T-cells infiltration, *in situ* perturbation

## Abstract

Tumor-infiltrating immune cells experience significant metabolic reprogramming in the tumor microenvironment (TME), and they share similar metabolic pathways and nutrient needs with malignant cells. This positions these cell types in direct nutrient competition in the TME. We currently lack a complete understanding of the similarities, differences, and functional consequences of the metabolic pathways utilized by activated immune cells from different lineages *versus* neoplastic cells. This study applies a novel *in situ* approach using implantable microdevices to expose the tumor to 27 controlled and localized metabolic perturbations in order to perform a systematic investigation into the metabolic regulation of the cellular fitness and persistence between immune and tumor cells directly within the native TME. Our findings identify the most potent metabolites, notably glutamine and arginine, that induce a favorable metabolic immune response in a mammary carcinoma model, and reveal novel insights on less characterized pathways, such as cysteine and glutathione. We then examine clinical samples from cancer patients to confirm the elevation of these pathways in tumor regions that are enriched in activated T cells. Overall, this work provides the first instance of a highly multiplexed *in situ* competition assay between malignant and immune cells within tumors using a range of localized microdose metabolic perturbations. The approach and findings may be used to potentiate the effects of T cell stimulating immunotherapies on a tumor-specific or personalized basis through targeted enrichment or depletion of specific metabolites.

## 1 Introduction

The alteration of cellular metabolism is a well-known determinant of cancer progression (Pavlova and Thompson, 2016). This phenotype is needed to sustain viability and anabolic demand due to high proliferation rates. Moreover, to meet the requirements of rapid growth, metabolic rewiring has a profound impact on the biochemical composition of the tumor microenvironment (TME), leading to nutrient depletion, variation of physiological pH, hypoxia and oxidative stress, which directly influence the recruitment and activity of tumor infiltrating lymphocytes (TIL) (Leone and Powell, 2020). Given the advancement of cancer immunotherapy, including immune checkpoint blockade inhibitors and chimeric antigen receptor T cells (CAR-T cells), a deep understanding of metabolic perturbation in the TME, as well as the metabolic competition between tumor cells and immune cells, are essential for preventing cancer immune evasion and ultimately developing improved combination treatments.

Tumors are known to be heterogeneous tissues composed of diverse cell types, with spatially distinct, and biologically and chemically diverse microenvironments, in which the interaction between malignant cells and immune cells is a key determinant of cancer progression (Egeblad et al., 2010). The immune population in tumors is composed of a range of cell types (Fridman et al., 2017) of which T-cells have become a primary target for intervention through approval and widespread use of checkpoint inhibitors in clinical oncology (Waldman et al., 2020). Functionally, T-cells are classified into two categories: either for being able to exert an antitumor response (effectors), or for being tumor permissive and promoting an immunosuppressive microenvironment. Effector immune cells belong to both the innate and adaptive responses of the immune system. Amongst the adaptive system, CD8^+^ T_eff_ cells are crucial for direct, antigen-specific, tumor cell killing through apoptosis induction and inflammatory cytokine secretion. Similarly, the immunosuppressive population is composed of cells belonging to both immune system branches, in which CD4+FOXP3+ T_reg_ cells constitute the lymphoid compartment. These cells have the physiological role of dampening the immunity response, but in the tumoral tissue they prevent the effectiveness of the antitumor action of the effector cell population.

Different immune cells can have very specific metabolic pathway regulation and critical metabolite dependencies, according to their state and function (Patel et al., 2019). Some of the major players in the metabolic machinery relevant for T cell activity include glucose and glycolysis, amino acids, tricarboxylic acid (TCA) cycle, lipids, and reactive oxygen species (ROS).

Current efforts in cancer immunometabolism have sought to understand how to address the differential metabolic requirements comparing effector T cells and tumor cells. However, these two populations share broad similarities in their metabolic phenotype (Andrejeva and Rathmell, 2017), leading to inevitable nutrient competition in the TME which is exacerbated by poor vascularization, as has been shown for glucose consumption (Chang et al., 2015). Specifically, a lack of glucose has been shown to dampen the inflammatory signaling mediated by mTOR and IFN-ɣ in the CD8^+^ population, leading to tumor progression (Chang et al., 2015) The glycolytic pathway is historically known to be typically high in proliferating cells(Warburg, 1956), and more recently analysis obtained from patient data reported an inverse correlation comparing upregulation of glycolysis genes in the tumor and T cell infiltration (Cascone et al., 2018). Moreover, metabolic waste compounds, such as lactate, the byproduct of intense glycolysis, has been shown to influence T_eff_ cells activity and infiltration, modulating the immune response in favor of cancer progression (Cascone et al., 2018). Indeed, high lactate levels and overexpression of the enzyme lactate dehydrogenase (*LDHA*) in the TME can suppress CD8^+^ cell proliferation, survival, cytokine secretion and cytotoxicity (Fischer et al., 2007; Brand et al., 2016). However, more recent works have highlighted other interesting roles of lactate in CD8+: an *in vivo* study has shown how lactate injection can increase stemness like features, increasing antitumor activity *via* the inhibition of histone deacetylase (Feng et al., 2022) and the mitochondrial pyruvate carrier has been linked to the maintenance of lactate oxidation to support antitumor function in CAR-T cells (Wenes et al., 2022). Overall, the role of lactate in tumor response is complex, and function may vary depending on tumor model and method of study.

Other metabolites can substantially influence persistence and function of the lymphocyte. Some of the most important are amino acids and their byproducts, which are crucial for incorporation into cellular biomass. Similarly to cancer cells, highly active T cells rely on amino acid metabolism to sustain intense protein and nucleotide synthesis. As such, the expression of several transmembrane transporters is highly upregulated upon T cell activation (Ren et al., 2017), sustained by the mTOR and MYC signaling pathway (Wang et al., 2011). Arginine constitutes a good example of an essential metabolite for correct T cell response, deprivation of this semi essential amino acid can lead to cell cycle arrest (Rodriguez et al., 2007). Arginine is the precursor for several molecules, including creatine and polyamine (Patel et al., 2019), and its supplementation improves T cell fitness and antitumor activity *in vivo* (Geiger et al., 2016). Tryptophan, is another amino acid with a central role in the immune system regulation. Kynurenine, has a physiological immunosuppressive role which can be exploited by cancer cells (Routy et al., 2016). Overexpression of indoleamine 2,3-dioxygenase (IDO) in the TME may lead to the suppression of the T cell antitumor response, depleting tryptophan and substituting it with kynurenine (M. Liu et al., 2018).

Glutamine is a highly studied amino acid in proliferating cells, given its contribution to both the carbon skeleton and nitrogens, to synthesis of amino acids, nucleic acids and lipids (Altman et al., 2016), through a process that replenish the tricarboxylic acid (TCA) cycle intermediates called anaplerosis. Both cancer cells and active T cells can be glutamine avid (Carr et al., 2010). In active T cells the expression of glutamine transporter (ASCT2, also called SLC1A5) is heavily upregulated to sustain the demand for this amino acid (Nakaya et al., 2014). Intriguingly, T cells are not strictly dependent on glutamine for their functionality, instead, TILs that undergo glutamine blockade are capable to overcome the potential stress condition upregulating pyruvate carboxylase (PC) (Leone et al., 2019). However, this phenotype can also be observed in cancer cells *in vitro* and *in vivo* (Cheng et al., 2011), suggesting that chronic suppression of glutamine metabolism can be efficient if PC activity is not upregulated in response. Lastly, the amino acid cysteine which is typically provided by macrophages and/or dendritic cells (DC) has also been shown to be essential for T cell survival, specifically in the TME.

With such a wide range of metabolic pathways and nutrient requirements shared between cancer cells and T-cells in the TME, the fundamental question remains as to how changes in the levels of key metabolites in the TME will impact the proliferative balance of these competing cell types. Most studies characterize the T cell sensitivity to a given molecule through *in vitro* experiments (typically by media supplementation or depletion) or *via in vivo* dosing, performed either with systemic administration or localized injection. Available *in vitro* and *ex vivo* systems have proven useful for investigating specific mechanistic questions, but the wide range of cell types that are present in a live tumor, and their spatial and temporal interdependence, cannot be recreated faithfully outside of the body as metabolite or gene expression levels change rapidly when tissue is removed from its native environment in the organism, leading to very different observed phenotypes (Davidson et al., 2016). Systemic dosing studies are difficult to conduct due to delivery challenges associated with selectively altering nutrient levels in tumors, and regional tumor heterogeneity (for instance, varying T-cell density at baseline) lowers the signal-to-noise ratio in whole tumor studies.

This study uses intratumor implantable microdevices to deliver locally and precisely tuned quantities of metabolites to confined regions of the tumor, in order to examine how the relative proliferation of tumor *versus* immune cells is altered in response to an increase in these nutrients. We then use a panel of targeted metabolic inhibitors to create local niches in the tumor with depressed levels of key metabolites, such that both an increase and a decrease in key metabolic pathway activity is examined. The study represents the first instance of delivery of 15 metabolites and 12 metabolic inhibitors *in situ* and in parallel within the same tumor, focusing on how local nutrient enrichment and depletion control the balance of T_eff_ cells and T_reg_ cells within the TME, aiming to characterize which perturbations are significant for both adaptive immune responses, and further delineating a specific signature typical of each T cell lineage.

## 2 Methods

### 2.1 Animal model

Institutional animal care and use committee (IACUC) approval was obtained. Murine spontaneous PyMT (Lin et al., 2003) (breast cancer) tumors were used for this study. Tumors were grown to 7–8 mm maximal diameter prior to microdevice insertion. Microdevices preloaded with drug and assay were directly inserted into live tumoral tissue. After the microdevice implantation, the preloaded microdoses of drugs passively diffused into spatially discrete tissue regions. Devices remained *in situ* for 7 days.

### 2.2 Implantable microdevice

Implantable drug delivery devices were manufactured as described previously (Jonas et al., 2015). In short, cylindrical microscale devices with dimensions 750 μm (diameter) × 6 mm (length) were manufactured from medical-grade Delrin acetyl resin blocks (DuPont) by micromachining (CNC Micromachining Center). Circular reservoirs (18 per device) were shaped on the outer surface of devices with dimensions 200 μm (diameter) × 250 μm (depth). 1-2 IMD can be inserted into the tumoral tissue.

The implantable screening microdevice (IMD) remains in the tumor for 7 days, where metabolites are released into a confined region of tissue adjacent to each reservoir. The tumors are processed for histological staining on several single fixed paraffin embedded (FFPE) tissue sections, each one corresponding to up to two drug delivery areas, thus allowing multiple quantitation of different cell types. This process allows performing single cell segmentation on a non-dissociated tissue, in which the spatial architecture is preserved.

### 2.3 Metabolites, drugs, and cytokines formulation

Drugs for this study were purchased in solid powder form Selleck Chem, Houston, TX, United States (BPTES, Sulfasalazine, Rapamycin A769662, SnMP, P7C3, Etomoxir, BSO, GSK2194069). AGI15280, AGI-519 and GNE-140 were purchased from Adooq Bioscience. All metabolites were purchased in solid powder form from Sigma-Aldrich.

Drugs and metabolites were formulated with 8,000 g/mol molecular weight polyethylene glycol (PEG) (Polysciences, Inc.) into a 25% *w/w* drug-PEG-8000 composite powder and were loaded into micro-reservoirs as described in (Jonas et al., 2015). Drugs are chosen based on specific role in the immune and/or cancer metabolism.

### 2.4 Tumor tissue preparation

Mice are euthanized by CO_2_ exposure. Tumor tissues are immersed in 10% formalin for 48 h and placed into 70% EtOH before standard paraffin processing.

Multiple paraffin-processed samples are embedded in a paraffin block and sectioned on a standard microtome. At the first reservoir level of the IMD (identifiable by visual confirmation) serial sections of 6 μm thickness are collected. Sectioning continues until the next IMD reservoir level is reached, where more sections are collected. This process continues until sections from all IMD levels are obtained.

### 2.5 Multi-color immunofluorescence staining and imaging

Prior to staining, antigen retrieval/Quench (Leica Biosystem, RE7113-CE, PH6 or PH9) was performed in the microwave for 10–30 min (Microwave research and applications Inc. Model: BP-093). Tumor sections are deparaffinized in a dry incubator for 30 min at 60°C (BOEKEL Scientific) and subsequently immersed with xylene solution following gradient ETOH solution for 5 min. The tumor sections are incubated with primary antibodies for 30 min at room temperature. Tumor sections are washed three times with TBST solutions and incubated with Novacastra polymer (Leica Biosystem, Cat. # RE7101-CE) for 10 min. After that, sections are washed with TBST buffer and peroxidase blocking solution is applied (Leica Biosystem, Cat# RE7101-CE). For multicolor staining, we incubated Alexa fluorophore for 30 min.

Slides were then rinsed with TBST buffer, followed by antigen retrieval/quench in microwave for multi-color staining for 10–30 min. Continuously, slides were treated with other antibodies and rinsed in DI water for 20 min. After that, the tumor sections were mounted with diamond mounting media and placed on an automated fluorescent slide scanner (Leica Aperio Versa) for imaging. Image analysis was performed according to (Ahn et al., 2021). The analysis has been optimized to maximize the signal/noise ratio. The drug diffusion areas in the tissue (ROI), have been expanded up to 1,200 µm from the device and have been divided in four sub-ROIs, each at a depth of 300 µm. This strategy allows to exclude potential false positive or tissue damages that could prevent a correct quantification of the marker positive cells, which is particularly relevant in this setting, being T cells not extremely abundant in the PyMT tumors. Internal controls are the empty reservoirs in the same IMD.

### 2.6 Statistical analysis

The data were analyzed using Prism 9 (GraphPad, United States). Data were reported as the mean ± S.E.M. and a *p*-value less than 0.05 was considered as statistically significant. Comparisons between two groups were made using two-tailed unpaired Student’s t tests. In the Pathway Enrichment Analysis p values have been adjusted for multiple comparisons. The number of mice used for each group was calculated to achieve a power of 90% resulting in *n* = 4–6 per group.

### 2.7 MALDI imaging experiments

The tumors used for MALDI imaging were snap frozen upon retrieval. Tissue was sectioned using a standard cryotome, and tissue slices of 20 μm in thickness. Samples were cryosectioned at 14 μm and thaw mounted on glass slides; serial sections were analyzed. The orientation of the specimens was shifted for serial sections analyzed in positive *versus* negative ion mode, with device location used to orient the sample in each serial section. Slides were then coated with HCCA (7 mg ml−1; 50% methanol, 0.1% TFA) or 9-aminoacridine (10 mg ml−1; 70% ethanol, 0.1% TFA) using a TM sprayer (HTX Technologies). Serial sections were prepared for imaging analysis in both positive and negative ion modes on a 7T solariX-XR FTMS (Bruker) equipped with a dual ESI/MALDI source, a SmartBeam II 2-kHz Nd:YAG (355-nm) laser and paracell. Samples were analyzed with a raster width of 125 μm in positive ion mode and 100 μm in negative ion mode in the mass range of 80–2,000 m/z at an acquisition size of 2 MW. Images contained 7,200–10,500 pixels. Data were visualized, and co-registration of H&E images was performed using FlexImaging 4.1. Compound identifications were made on the basis of accurate mass (<1 ppm difference from expected mass) and isotopic peak matching.

### 2.8 Spatial transcriptomics

The GeoMx platform (Nanostring, Seattle United States) was utilized to quantify spatial gene expression from FFPE lung tissue sections. After preparation of the sections (baking, antigen retrieval, blocking), a multi-plex cocktail of profiling antibodies barcoded with photocleavable and uniquely indexed oligonucleotides were incubated on the tissues.

Regions of interest (ROIs) on FFPE slides were selected based on their positive index ratio of CD8^+^ positive cells/nuclei. After ROI selection, the GeoMx was used to UV-photocleave and collect oligonucleotides from the profiling antibodies staining the tissue, which were then deposited into a 96 well collection plate. Each ROI was illuminated independently to enable spatially resolved data acquisition. Oligonucleotides were then hybridized to fluorescent barcodes and quantified with the nCounter (Nanostring) Analysis System. The raw digital counts for the oligonucleotides were calibrated for oligo-barcode binding by the GeoMx. The data was then normalized to spike-in positive controls to assess data quality. The calibrated and spike-in normalized expression data was then normalized at a per ROI level to the geometric mean of the target group to enable the study of downstream differential expression.

## 3 Results

### 3.1 Metabolic perturbation of the tumor microenvironment identifies nutrients favorable for specific T cell populations

**FIGURE 1:**
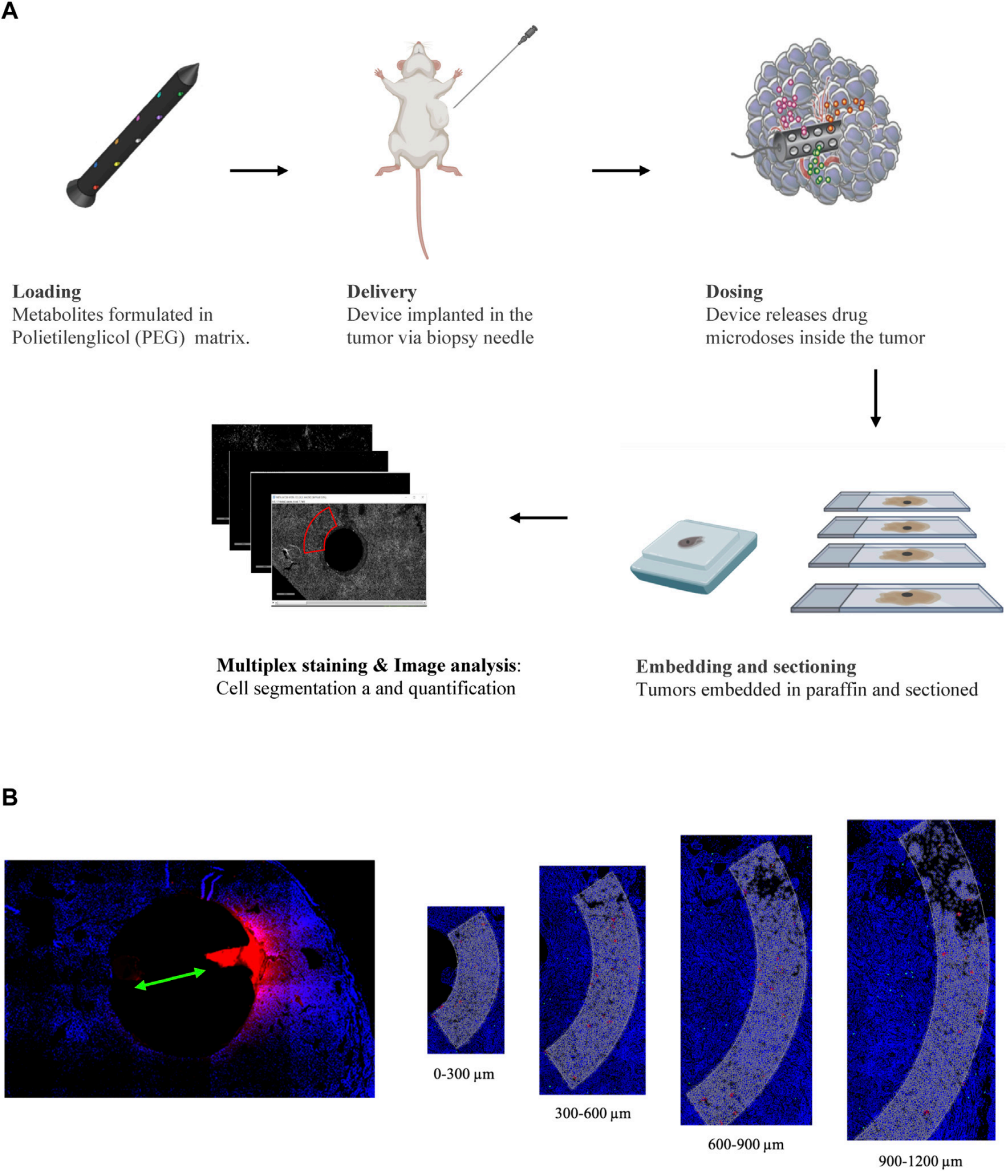
**(A)** Schematic representation of the experimental pipeline showing the different steps from IMD preparation to image analysis. **(B)** Image analysis workflow: drug release reservoirs are identified and ROIs are divided in 4 parts with 300 µm depth each.

We developed an experimental pipeline to dissect specific metabolite perturbation of the TME in a murine breast cancer model (Figure 1). We used an implantable screening microdevice (Jonas et al., 2015; Davidson et al., 2017) to evaluate the influence of each molecule of interest on different T-cell populations through multiplex immunohistochemistry (IHC)/immunofluorescence (IF) on tissue sections. To perform our initial screening, we chose 15 metabolites belonging to the major macromolecule classes already known to either promote or inhibit immune cell response, specifically in the TME (Table 1). We stained for some of the most important markers of the T cell population: CD3 (for all T cells), CD8 (for T_eff_ cells) and FOXP3 or T_reg_ cells). Studies were conducted in the oncogene driven immunocompetent MMTV-PyMT (mouse mammary tumor virus-polyoma middle tumor-antigen) breast cancer mouse model, characterized by late stage spontaneously growing tumors (Guy et al., 1992). MMTV-PyMT mice spontaneously develop mammary tumors that closely reproduce the progression and morphology of human breast cancers. Moreover, these tumors have been shown to elicit a robust host immune response, with detection of resident lymphoid cells in the TME (Dadi et al., 2016).

**TABLE 1:** Metabolites delivered with the bio microdevice in the MMTV-PyMT mice tumors.

Metabolite	Type	Role in cancer-Immuno-metabolism	Methods of study
Arginine	Amino Acid/Amino Acid metabolism product	High level enhances T cell antitumor response Geiger et al. (2016), T cell are more sensitive to its depletion in the TME Crump et al. (2021)	*In vitro* and *in vivo* through oral administration
Cysteine		Necessary for T cell activation Garg et al. (2011); Srivastava et al. (2010) and for anti-tumor response Lancien et al. (2021) limiting metabolite for glutathione synthesis Gmünder et al. (1990)	*In vitro/ex vivo*
Glutamine		Uptake increases upon T cell activativation (Carr et al. (2010), and it’s essential for the inflammatory response Nakaya et al. (2014)	*In vitro* and *In vivo* through labeled infusion
Methionine		Cancer cells and CD8^+^ T cells compete for the uptake. Supplementation favors anti-tumor immunity Bian et al. (2020)	*In vitro* and *In vivo* through intra-tumor injection
Kynurenic Acid		Catabolism product of tryptophan, generated through the activity of Indoleamine 2,3-dioxygenase 1 (IDO1) Labadie et al. (2019). Favors a tumor permissive microenvironment with PD-1 expression Liu et al. (2018) and CD4^+^ exhaustion Rad Pour et al. (2019)	*In vitro* and *In vivo* through i.p. injection
Tryptophan		Essential for T cell proliferation and activity Munn et al., (1999)	*In vitro/ex vivo*
Adenosine	Nucleoside/Nucleotide	Captured by the A2A receptor suppresses antitumor T cells Leone et al. (2018); Ohta et al. (2006)	*In vitro/ex vivo*
Uridine		Released with other pyrimidines by Tumor Associated Macrophages (TAM), impairs chemotherapy efficacy Halbrook et al., (2019)	*In vitro/ex vivo*
ATP		Extracellular ATP is converted in adenosine by receptors highly expressed by T_reg_ Deaglio et al., (2007), mediating immune suppression	*In vitro/ex vivo*
MTA		Induces T cell exhaustion and inhibits CD8^+^ antitumoral activity Henrich et al. (2016); Hung et al. (2021); Strobl et al. (2020)	*In vitro/ex vivo*
Hemin	Porphyrin	Inducer of heme oxygenase-1 (HO-1). Induce T cell antitumor response in prostate cancer Jaworski et al. (2017). Conversely, promotes immunosuppressive programs in TAM Alaluf et al. (2020)	*In vitro* and *In vivo* through sub cute injection
Palmitate	Fatty Acid	Relevant for metabolism of T_reg_, which rely both on glycolysis and fatty acids for oxidative processes Pacella et al., (2018)	*In vitro* and *In vivo* through i.p. injection
Sodium Pyruvate	Glycolysis & Tricarboxylic Acid (TCA) Cycle	Crossroad metabolite between Glycolysis and TCA. *In vivo* T_eff_ cells display preference for TCA vs. the glycolytic phenotype Ma et al., (2019)	*In vitro*/*In vivo* through labeled glucose infusion
Sodium Lactate		Suppression of proliferation and cytotoxic function of CD8^+^ T cells Fischer et al. (2007). Inhibition of immune surveillance in tumors Brand et al. (2016)	*In vitro/ex vivo*
Itaconate		Anti-inflammatory effects in macrophages Lampropoulou et al. (2016) and positively associated with tumor permissive immune response Weiss et al. (2018)	*In vitro/ex vivo*

**FIGURE 2:**
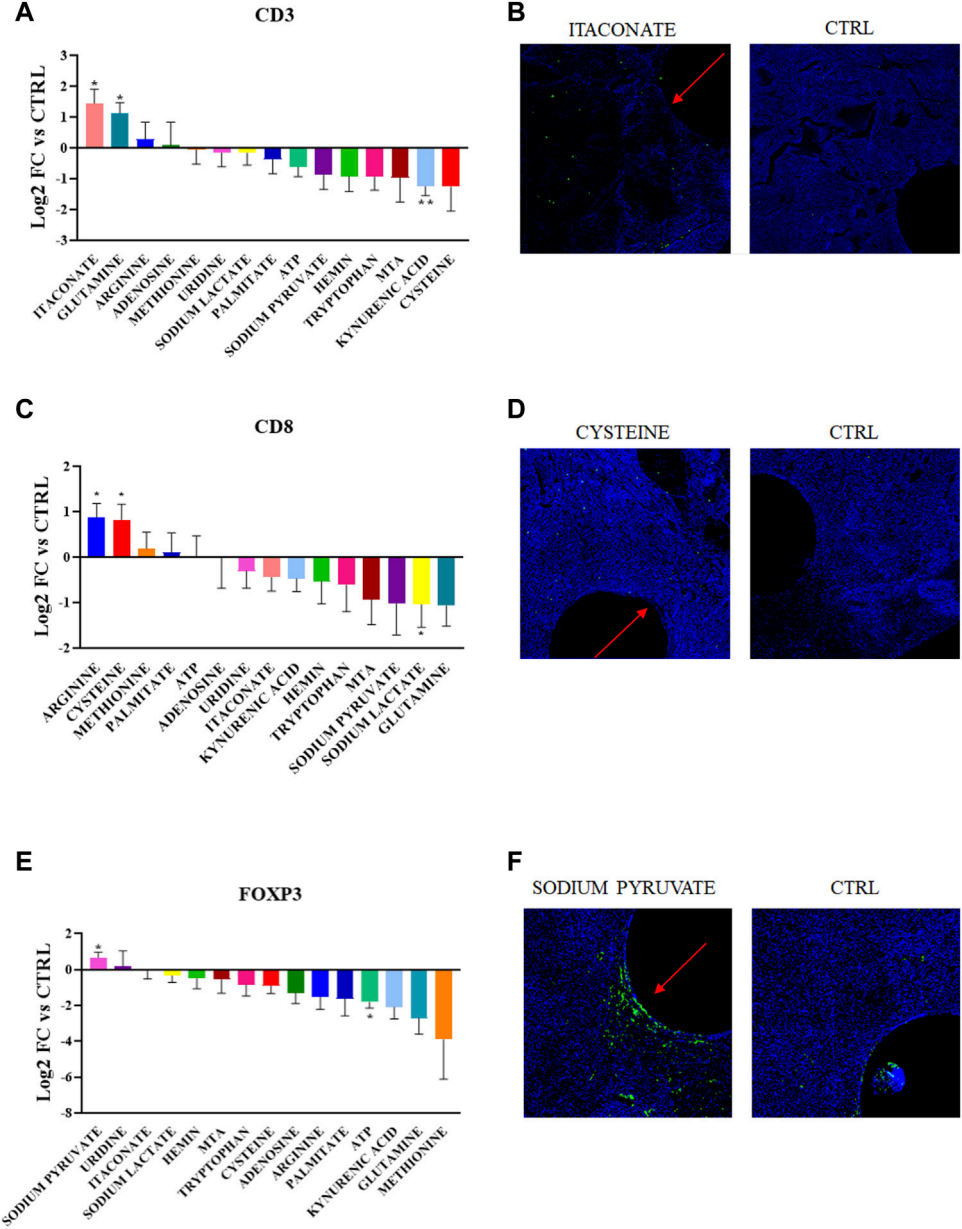
**(A,C,E)** Quantification of CD3^+^, CD8^+^ and FOXP3+ abundance in the corresponding region of each metabolic perturbation. Values are the Log_2_ of the fold change normalizing the number of positive cells detected in the ROI vs. positive cells in the control region of the same tumor. *p*-values indicate comparison between signal intensities at each drug site compared to Control (*p < 0.05, **p < 0.005) **(B,D,F)** Representative images showing the IF/IHC staining on the tumor sections.

Using this approach, we detected distinct effect of specific metabolites on each of the populations analyzed (Figure 2). The most notable effects were observed by local enrichment of the amino acids arginine and cysteine which lead to a statistically significant higher concentration of the CD8^+^ T cells in the TME in proximity to the device with an increase of 75%. Conversely, the release of sodium lactate in the tumor tissue significantly lowers CD8^+^ T cell presence by 50% compared to the control (Figure 2C, D).

Moderate increases in CD8^+^ T cell density were observed for methionine, palmitate and ATP, and glutamine and MTA release led to a decrease. Though none of these changes were statistically significant compared to the control. Though previous results obtained from *in vitro* studies for these molecules have identified metabolic dependencies, this represents the first direct *in situ* comparison of nutrient enhancement on T cell state.

Furthermore, direct release of sodium pyruvate in the TME favors the persistence of FOXP3+ T cells (60% increase), while extracellular ATP seems to have an overall negative effect (70% decrease). The CD3^+^ marker responds positively to the delivery of glutamine and itaconate (both metabolites induce an increase of 75%). While the metabolic significance of glutamine for T cells has been widely reported, itaconate has an unknown role in the lymphoid population and is involved in the anti-inflammatory phenotype in macrophages. T cells are also negatively regulated by kynurenic acid, which is in accordance with past studies (Rad Pour et al., 2019).

### 3.2 *In situ* delivery of metabolic inhibitors identifies pathways affecting abundance of different T cell populations

**FIGURE 3:**
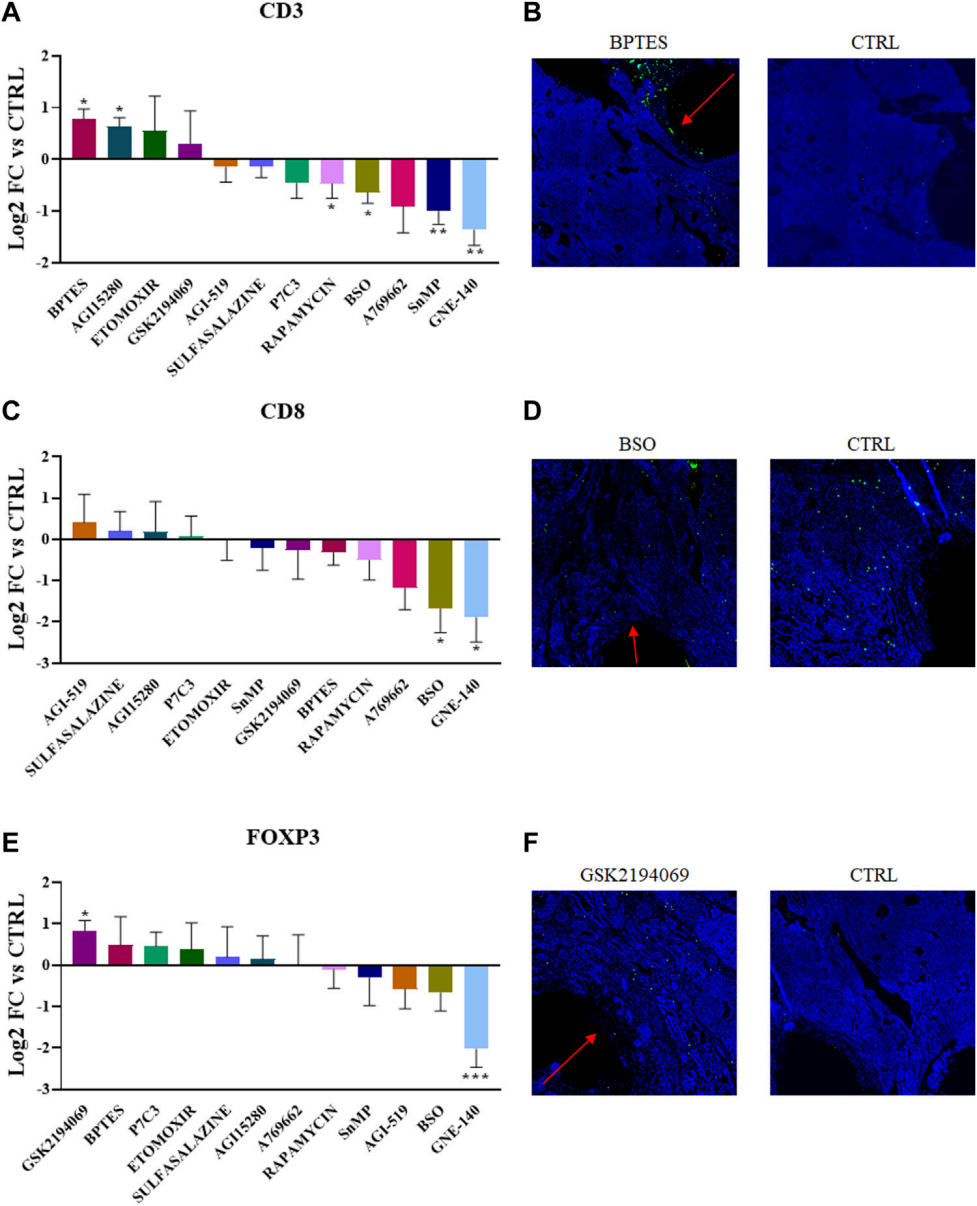
**(A,C,E)** Quantification of CD3^+^, CD8^+^ and FOXP3+ abundance in the corresponding region of each drug perturbation. Values are the Log_2_ of the fold change normalizing the number of positive cells detected in the ROI vs. positive cells in the control region of the same tumor. *p*-values indicate comparison between signal intensities at each drug site compared to Control (*p < 0.05, **p < 0.001 ***p < 0.0001) **(B,D,F)** Representative images showing the IF/IHC staining on the tumor sections.

We tested 12 targeted inhibitors that induce well-defined metabolic perturbations in the TME with the use of drugs targeting relevant pathways either in the cancer or immune landscape. When possible, we choose to target similar pathways observed in the nutrient-delivering device, to gain a deeper understanding of the importance of a particular metabolites *in situ*. Inhibitors (full list in Table 2) were delivered and their effect on the T cell populations was observed after 7 days of intratumor exposure (Figure 3). The inhibitor of LDH, GNE-140, significantly decreases all three T cell markers analyzed (with an average of 75%), an effect that might be an indicator of general toxicity of the drug towards lymphoid cells. Rapamycin, a known immunosuppressant and inhibitor of protein and lipid synthesis mediated by mTOR, was observed to have a negative effect *in situ* which was statistically significant for all markers including CD3. Interestingly, both glutaminolysis inhibitors (BPTES, and AGI15280) show an increased amount of CD3^+^ cells in their delivery areas, suggesting that glutamine in the TME is not needed for anaplerotic processes (to be converted to glutamate and then to ɑ-ketoglutarate). Moreover, the glutathione synthesis inhibitor, BSO, decreases both CD3^+^ and CD8^+^ cells by 50% and 75% respectively, suggesting that tumor infiltrating lymphocytes are particularly sensitive to glutathione deprivation. This effect on the redox imbalance is further confirmed by direct treatment with SnMP, which negatively impacts CD3^+^ abundance. GSK2194069, a fatty acid synthesis inhibitor, is the only drug which creates a favorable condition for FOXP3+ cells, confirming the metabolic plasticity of T_reg_ cells, as opposed to CD8^+^ cells with antitumor activity. These data demonstrate differential effects on T cell abundance based on the specific metabolic pathway which was perturbed, with the most potent agents being those affecting amino acid metabolism and redox balance.

**TABLE 2:** Drugs targeting cell metabolism delivered with the bio microdevice in the MMTV-PyMT mice tumors.

Drug	Mechanism of action	Effect on TME (cancer and immune cells)	Methods of study
BPTES	Inhibitor of the non-liver glutaminase isoform (GLS) Robinson et al. (2007)	Inhibits proliferation on cancer cells relying on glutaminolysis Thangavelu et al. (2012) (mitochondrial conversion of glutamine to glutamate Curthoys and Watford (1995). Unknown on T cells	*In vitro* and *in vivo*
AGI15280	Inhibitor of GLS van Gastel et al. (2020)	Comparable to BPTES van Gastel et al. (2020)	*In vitro* and *in vivo*
Sulfasalazine	Inhibitor of the x_c_^−^ cystine/glutamate antiporter Gout et al., (2001)	Antitumor activity on cancer cells that cannot synthesize cysteine, such as lymphoma Gout et al. (2003). Can induce Macrophages M2 polarization in melanoma Liu et al. (2021) Unknown on anti-tumor T cells	*In vitro* and *in vivo*
Rapamycin	Immunosuppressant known to bind and inhibit the mechanistic target of rapamycin (mTOR) Sabatini et al., (1995), which controls several processes in the cell, including protein and lipid synthesis Laplante and Sabatini, (2012)	Despite mTOR being essential for antitumor CD8^+^ memory T cell development Araki et al. (2009); Li et al. (2011), p. 8), systemic treatment with rapamycin impairs CD8^+^ response Chaoul et al. (2015). Mouse models with persistent mTOR activation showed impaired tumor growth Pollizzi et al. (2015)	*In vitro* and *in vivo*
A769662	Activator of AMP-activated protein kinase (AMPK) which is a key sensor and regulator of intracellular and energy metabolism	Demonstrated synergy with checkpoint blockade inhibitors, improving immunotherapy and T cell anti-tumor activity in mouse models Chamoto et al. (2017); Dai et al. (2021)	*In vitro* and *in vivo*
Buthionine Sulfoximine (BSO)	Glutathione synthesis inhibitor Griffith and Meister, (1979)	Induces macrophages immunosuppressive phenotype because of higher concentration of reactive oxygen species (ROS) Roux et al., (2019) Unknown on tumor infiltrating T cells	*In vitro* and *in vivo*
P7C3	Activator of the nicotinamide phosphoribosyltransferase (NAMPT), involved in NAD production Wang et al., (2014)	Discovered as a neuroprotective chemical Pieper et al. (2010), inhibited malignant growth of glioma, targeting phosphoglycerate kinase 1 (PGK1).Chen et al. (2021)	*In vitro* and *in vivo*
SnMP	Inhibitor of HO-1 activity Chernick et al., (1989)	Inhibits the immune suppression of CD8^+^ T cell by targeting myeloid HO-1 activity in the TME in MMTV-PyMT tumors Muliaditan et al., (2018)	*In vitro* and *in vivo*
Etomoxir	Irreversible inhibitor of carnitine palmitoyltransferase 1 (CPT1) Kiorpes et al. (1984)	Fatty Acid Oxidation (FAO) inhibition blocks immune inhibitory pathways in myeloid-derived suppressor cells (MDSC) and promotes antitumor T cell activity Hossain et al. (2015). In T cells, it has been shown to induce severe oxidative stress independent from FAO O’Connor et al. (2018)	*In vitro* and *in vivo*
GSK2194069	Inhibitor of the β-ketoacyl reductase (KR) activity of human fatty acid synthase (FAS) Hardwicke et al. (2014)	Inhibits cell growth in some cancer cells Hardwicke et al. (2014), but its effect looks associated with general toxicity in other cell lines Singha et al. (2020). Unknown on immune cells	*In vitro*
AGI-519	Pyruvate kinase-R (PKR) activator	The molecule has been withdrawn from further studies by Agios Pharmaceuticals (Agios Provides Update on PKR Program)	*In vitro*
GNE-140	Lactate dehydrogenase (LDHA and LDHB) inhibitor Boudreau et al. (2016)	Slows tumor proliferation on cells relying on aerobic glycolysis. (Boudreau et al. (2016); Ždralević et al. (2018)	*In vitro* and *in vivo*

### 3.3 Metabolomic analysis in the tumor microenvironment reveals a unique metabolic signature specific to the T cell population

**FIGURE 4:**
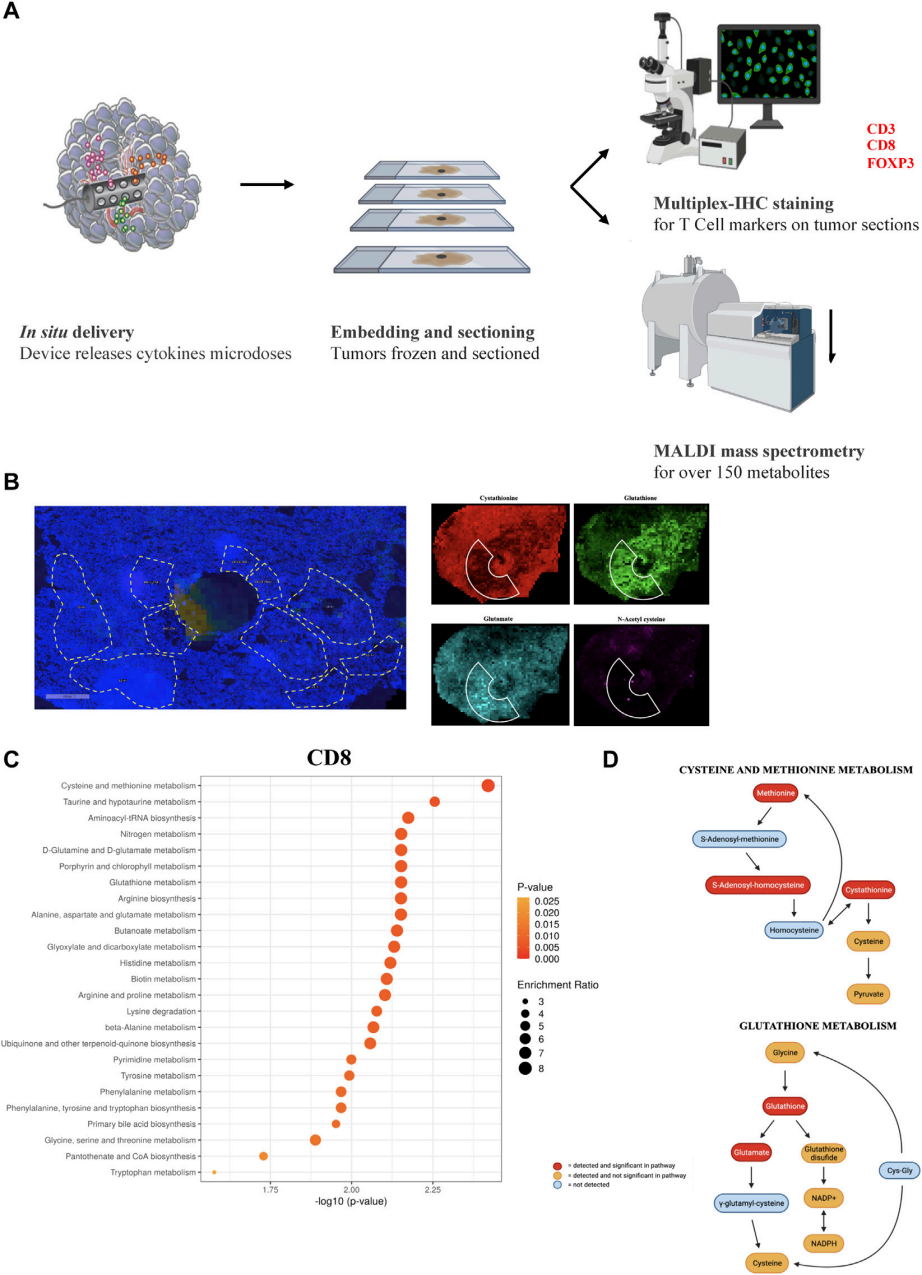
**(A)** Schematic representation for the unbiased metabolomics experiment. **(B)** Representative images showing the ROIs selection and metabolite quantification. **(C)** Top 25 most significant pathways enriched comparing CD8 high vs. CD8 low ROIs. **(D)** Statistical significance of single metabolites in the Cysteine and Methionine Metabolism and Glutathione Metabolism pathways (significant values have *p* < 0.05).

To confirm that the compounds which showed either a positive or negative effect on the various T cell subpopulations are intrinsically relevant for the metabolism and persistence of the targeted cell types, we reversed our approach and created enriched immune cell regions within tumors which were then analyzed using spatial tumor tissue metabolomics. First, we reproduced similar biological conditions to the previous experiments by direct cytokine release (Figure 4A). A selected group of cytokines (IL-2, IFN-ɣ, CXCL9, ICAM-1, and GM-CSF) were loaded and released in the PyMT tumors for 7 days, with the goal of inducing an enriched region of higher immune cell density around the microdevice area. Following tumor retrieval and frozen sectioning, serial tissue sections were analyzed using a dual modality approach: multiplex IF for immune cell markers and MALDI mass spectrometry for measuring spatial abundance of 160 metabolites. Regions of interest (ROIs) with high T cell specific marker abundance were identified, and each cluster of CD3^+^, CD8^+^ or FOXP3+ abundance was coupled with a nearby ROI of equivalent area, with at least 0.5-fold change cells of the same maker. This allowed us to directly compare proximate areas of tumor with distinct immune cell biology (Figure 4B), and extract raw ion counts for each metabolite quantified in each ROI to obtain a direct comparison between those TME metabolic conditions favoring immune *versus* tumor cell proliferationWe compared 37 matched pairs of adjacent ROIs for CD3, 45 pairs for CD8, and 34 pairs for FOXP3, measuring relative metabolite abundance and pathway enrichment analysis with Metaboanalyst, a web-based interface for metabolomics data analysis (Xia et al., 2009; Chong et al., 2019; Pang et al., 2021). We determined that CD8^+^ regions have several pathways significantly enriched, including, most notably, cysteine, methionine and glutathione metabolism (Figures 4C, D). These data which were identified using an unbiased data mining approach, are in agreement with our previous findings showing CD8^+^ cells being positively affected by cysteine delivery and negatively impacted by BSO release in the TME. CD3^+^ and FOXP3+ analysis did not show statistically significant pathway enrichment, which might be due to the lower number of ROIs available for comparison (Supplementary Figures S1A,B).

**FIGURE 5:**
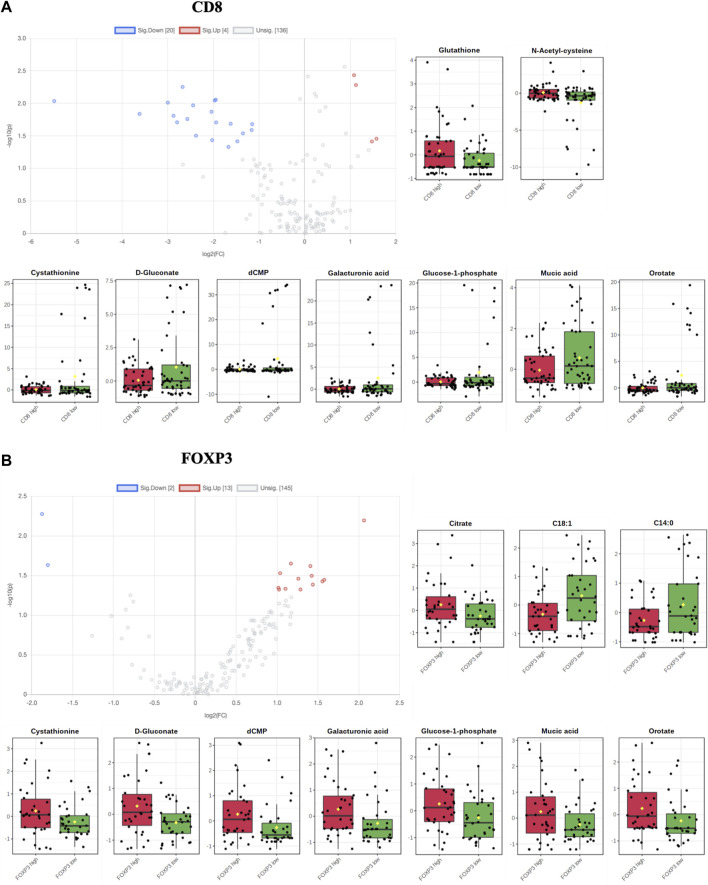
**(A,B)** Volcano plots and single metabolite enrichment representation for both CD8 and FOXP3 enriched ROIs (significant values have *p* < 0.05).

To further determine the impact of specific T cell states on TME metabolomics, we plotted single metabolite enrichment for each marker. We observed that Glutathione and N-acetyl-cysteine (NAC), two essential antioxidant molecules, are significantly enriched in CD8 high ROIs (Figure 5A; Supplementary Figures S2A). On the contrary, FOXP3 high ROIs showed higher citrate, which is implicated in TCA and fatty acid metabolism, and lower abundance of two lipids, myristic acid and oleic acid (Figure 5B; Supplementary Figures S2B).

Moreover, we noticed that seven metabolites (cystathionine, d-Gluconate, dCMP, galacturonic acid, glucose 1-phosphate, mucic acid and orotate) showed opposite trends in the CD8^+^ *versus* the FOXP3+ population, indicating a characteristic metabolic signature of th antitumor and protumor lymphoid populations based on their specific metabolic needs.

**FIGURE 6:**
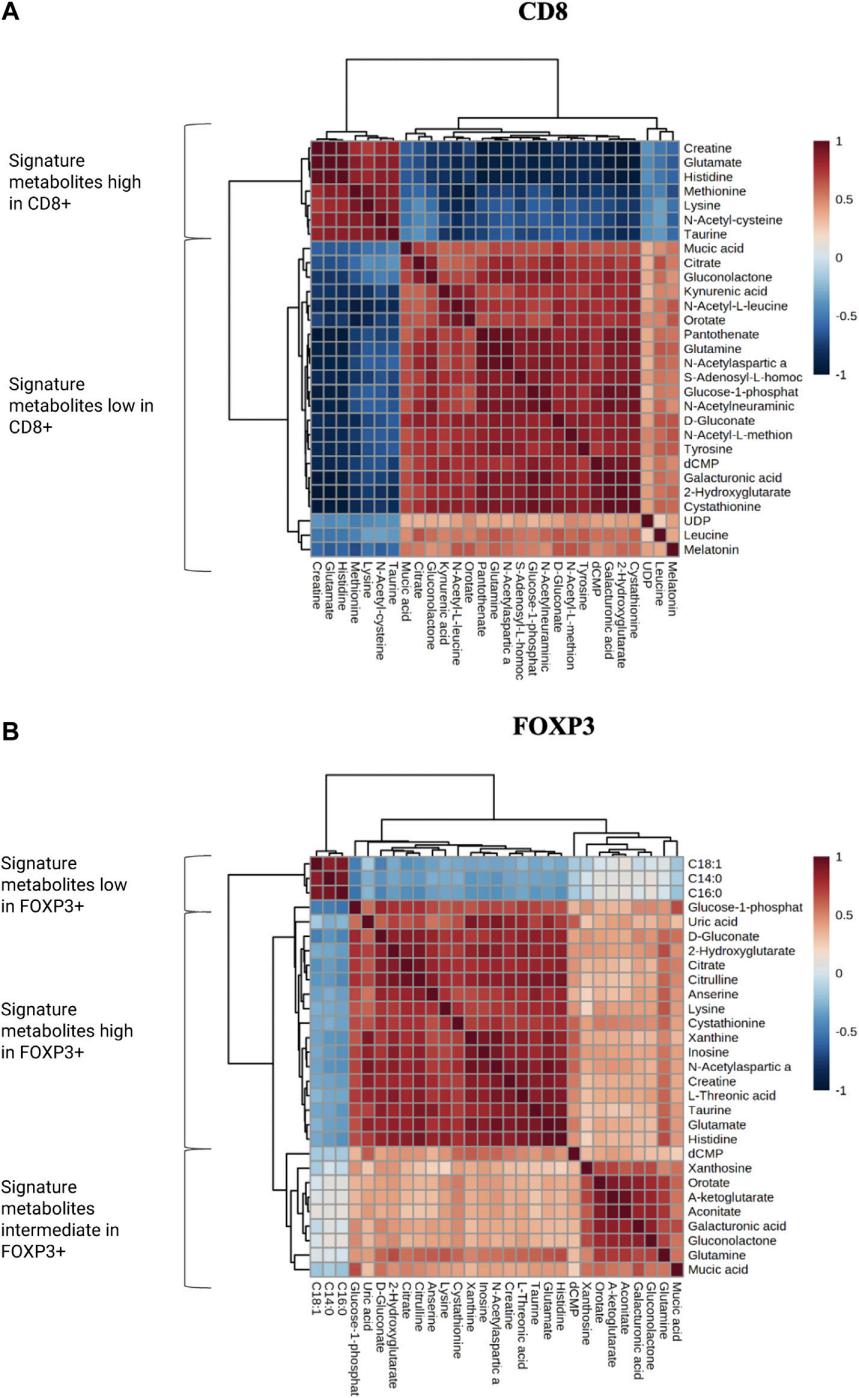
**(A,B)** Correlation matrixes for the most and less enriched metabolites in both CD8^+^ and FOXP3+ ROIs. Metabolites have been categorized in low, high and intermediate according to their abundance.

Notably, we observed that glutamate, creatine and histidine, showed opposite correlation patterns in CD8^+^ significant metabolites *versus* FOXP3+ significantly varying metabolites (Figure 6). We further selected the most relevant metabolites displaying significant variation in the two populations and we identified specific clusters which are characteristic of either CD8^+^ or FOXP3+ T cells, creating a “metabolomic signature” specific to their recruitment in TME. Specifically, these clusters exhibit a significant inverse correlation pattern. For instance, we observed that citrate, glutamine, kynurenic acid and cystationine are consistently present in lower amounts in CD8^+^ enriched regions, while some fatty acid chains such as C18:1, C16:0 and C14:0 are found to be reduced in FOXP3+. We hypothesize that this signature could be further exploited to understand if the tumor tissue is prone to favor T_eff_ vs. T_reg_.

### 3.4 Spatial transcriptomics analysis on human lung adenocarcinoma samples shows similar metabolic pathway signature for CD8 enriched regions

**FIGURE 7:**
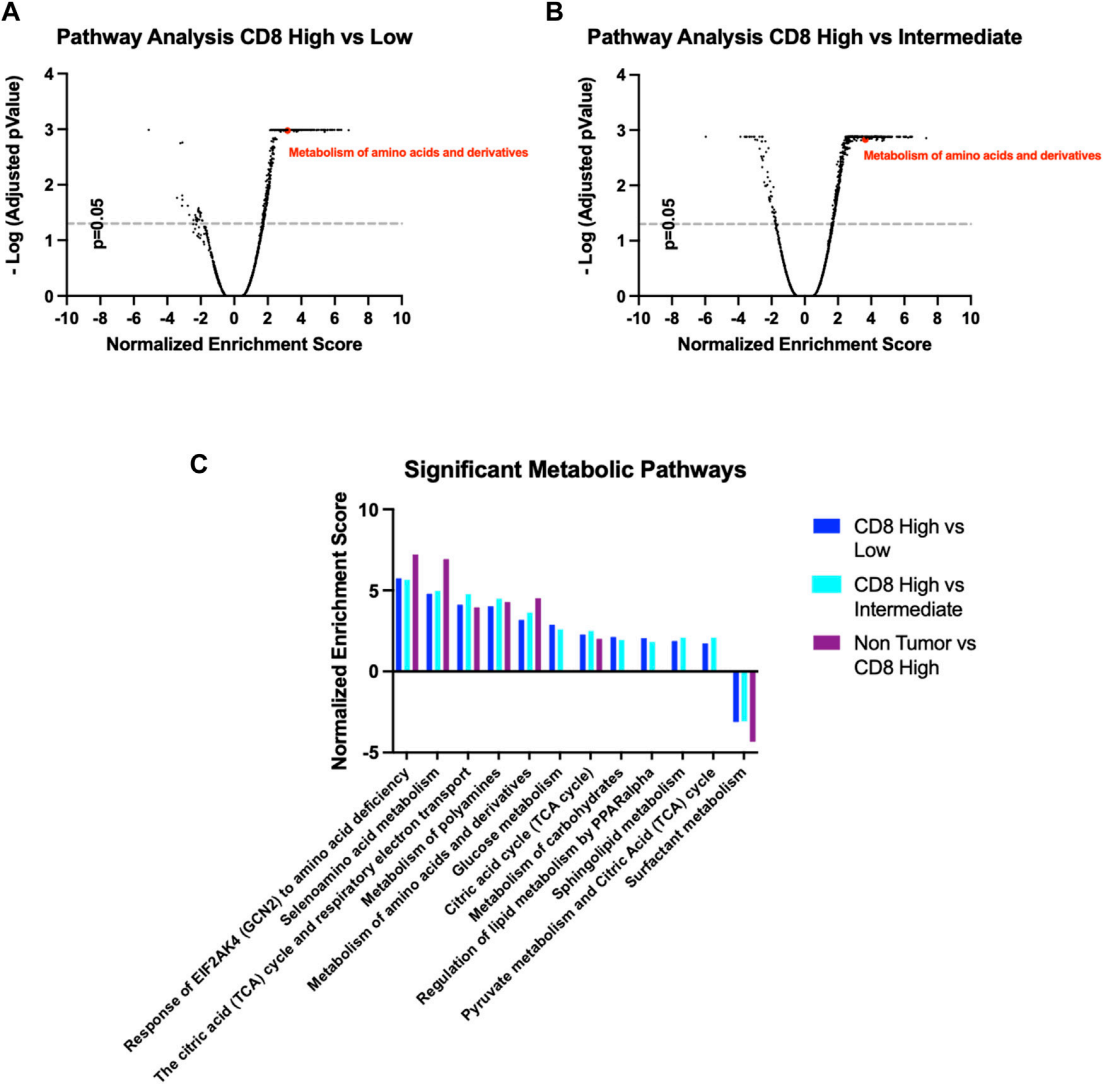
**(A,B)** Volcano plots for pathway enrichment in the Reactome Pathway Database comparing CD8 high with either CD8 intermediate or CD8 low ROIs. **(C)** Significant metabolic pathways follow similar pattern according to their CD8 infiltration, CD8 high ROIs being the most similar to the non-tumoral tissue.

We sought to confirm the previous observations made in murine tumors regarding tumor infiltrating CD8 metabolic preference for specific pathways, in human samples. Nanostring spatial transcriptomic analysis was performed on human lung adenocarcinoma samples based on where breast cancer lesions metastasize, consistent with our mouse model PyMT. IF staining against CD8 on several sections of the FFPE tissue identified regions with different levels of T-cell recruitment. We identified 30 different ROIs which were classified in CD8 high (positive index >20%, 9 count), CD8 intermediate (5% < positive index >20%, 12 count) and CD8 low (positive index <5%, 9 count). Whole-transcriptome sequencing in the aforementioned ROIs was performed to measure the expression of more than 18500 genes across ROI cohorts. Pathway analysis comparing the three different classes of ROIs was performed to understand how metabolism changes according to CD8^+^ T cell infiltration (Figure 7). Interestingly, several metabolic pathways are shown to be enriched in CD8^+^ high ROIs compared to both CD8^+^ intermediate and CD8^+^ low ROIs, such as glucose metabolism, pyruvate metabolism, TCA cycle and respiratory electron transport, which aligns with the high energetic demands required by active T_eff_ cells engaging anti-tumor activity.

We also found the Reactome Pathway Database “Metabolism of amino acids and derivatives” (ID: R-HSA-71291.6) to be significantly enriched, in accordance with our previous data from PyMT mice (Figures 7A,B). Importantly, this metabolic pathway signature displays an identical trend when comparing Non-Tumor tissue to CD8^+^ high ROIs, indicating these pathways are indicative of either anti-tumoral or non-tumoral cellular activity (Figure 7C). Overall, these data confirm our hypothesis that T-Cell mediated infiltration in the TME is marked by specific metabolic features, that can be selectively induced by intratumor nutrient enhancement or targeted inhibition.

## 4 Discussion

This study describes the first *in vivo* direct comparison of the effect of 27 parallel metabolic perturbations on persistence of T cells in the native TME. The metabolism of amino acids and their derivatives is a highly studied pathway of immune cell activation and tumoral cell growth. To date, glutamine and arginine are two molecules that have been characterized both *in vitro* and *in vivo* in the context of the T cell antitumor response, through labeled infusion or enteral administration. Our findings direclty confirm the role of these amino acids in the TME, and provide direct evidence of the essential role of cysteine metabolism in the recruitment of CD8^+^ T cells in the tumoral tissue *in vivo* and *in situ*. We observed enrichment in CD8^+^ T cells upon delivery of cysteine in the tumoral tissue, but no significant effect with delivery of sulfasalazine, the inhibitor of the x_c_^−^ cystine/glutamate antiporter (Gout et al., 2001). While it is known that naïve T cells are dependent on antigen presenting cells (APCs) to provide cysteine (Gmünder et al., 1990; Angelini et al., 2002), it has also been demonstrated that activated CD4^+^ T cells are able to upregulate the gene expression of x_c_^−^, rendering them independent from the APCs (Levring et al., 2012). Still, the role of x_c_^−^ *in vivo* is not well understood: one study found that the transporter was dispensable for T cell proliferation *in vivo* and for the immune responses to tumors (Arensman et al., 2019). Our results confirm these findings, indicating that the role of cystine transporter in T cells warrant further investigation. The cytoplasmic concentration of cysteine is essential for glutathione synthesis (Siska et al., 2016), which is the main reducing agent counteracting reactive oxygen species (ROS), which are often the product of increased metabolic activity and which can be toxic when accumulated in excess. Indeed, we observe an effect similar to cysteine starvation when we deliver buthionine sulfoximine (BSO), a specific inhibitor of the glutathione biosynthesis (Gmünder et al., 1990).

Our study further demonstrates the relevance of both cysteine and glutathione pathways *in vivo* based on an unbiased metabolomic screen, where we take advantage of the fact that T cells accumulate spontaneously in spatially distinct regions, with the underlying hypothesis being that such regions feature a metabolic microenvironment which is favorable to such cells. These selected regions of interest that are based on local CD8^+^ T Cell abundance, exhibit significant single metabolite enrichment for glutathione and N-acetyl-cysteine, both molecules with antioxidant properties. Analogously, CD8^+^ compared to FOXP3+ cells display different enrichment patterns for specific metabolites, outlining a characteristic “metabolic signature” of pro-tumor or anti-tumor metabolic environment characterizing the T cell response in the TME. Some of these signature metabolites have been already widely characterized (e.g., glutamine) which we detected consistently in lower abundance in CD8^+^ enriched areas. Others, such as histidine, which we found enriched in CD8^+^ ROIs, do not have a well-defined role in the anti-tumor activity of T_eff_ cells. In future studies we hope to test whether these metabolic patterns could be exploited to identify favorable vs. adverse TME conditions as a predictive marker for immunotherapy efficacy in patients. Some of the identified metabolites (e.g., cysteine and glutathione) were shown to be important for the fitness and most importantly the antitumoral activity of CD8^+^ T cells. To the best of our knowledge, this is the first time that their role is demonstrated directly in the tumor tissue *in vivo* and *in situ*, maintaining the spatial integrity and composition of the native tissue. Lastly, we used available human tumor samples to perform spatial transcriptomic analysis, and observed the same trend of specific metabolic pathway enrichment, most notably in amino acid metabolism and derivatives, are seen as directly correlated with CD8^+^ T cell infiltration in the actively treated tumor tissue.

A limitation of the current study is that a time point of 7 days was chosen as the endpoint, as technical limitations due to the need of keeping nutrient diffusion zones spatially separated currently prevent longer in-dwelling times for the microdevice. It is possible that at longer time points, other nutrients may show additional T cell enrichment, though it appears unlikely that this would significantly alter the relative impact of different nutrients.

Another challenge consists in determining if the T cells identified through multiplex immunohistochemistry (IHC)/immunofluorescence (IF) are active or quiescent. Unfortunately, most activation markers are either cytokines or phosphorylated proteins, which are difficult to identify on fixed paraffin embedded (FFPE).

Overall, this work is the first to directly measure the effects of altering nutrient competition in a native tumor system for multiple nutrient and metabolic pathway perturbations in parallel, and identifying those metabolic perturbations that are most amenable to CD8^+^ cytotoxic T cell aggregation. At a technical level, the IMD approach may be useful to rapidly screen many metabolic perturbations in a native tissue system. Biologically, since baseline T-cell infiltration is an important marker associated with the efficacy of immunotherapies, the findings made in this study, for instance related to cysteine metabolism, may be used to further investigate combination treatments consisting of immunotherapies and metabolic modulators which would be anticipated to lead to higher response rates. One key metabolite implicated in this study for CD8^+^ cell persistence is cysteine, and elevating its intratumor levels may be exploited to both understand CD8^+^ favorable conditions in order to create pro-inflammatory and anti-tumoral activity, and to develop more potent therapeutic combinations with checkpoint inhibitors. We envision two distinct approaches to achieve this goal: targeting pathways that improve the antitumor immune cell persistence and function without positively interfering with cancer cell proliferation and survival; or targeting pathways that are essential for cancer cell survival but redundant to the effector immune cell function.

## Data Availability

The original contributions presented in the study are included in the article/supplementary material, further inquiries can be directed to the corresponding author.

## References

[B1] Ahn S. W. Ferland B. Jonas O. H. (2021). An interactive pipeline for quantitative histopathological analysis of spatially defined drug effects in tumors. J. Pathol. Inf. 12, 34. 10.4103/jpi.jpi_17_21 PMC8529341 34760331

[B2] Alaluf E. Vokaer B. Detavernier A. Azouz A. Splittgerber M. Carrette A. (2020). Heme oxygenase-1 orchestrates the immunosuppressive program of tumor-associated macrophages. JCI Insight 5, 133929. 10.1172/jci.insight.133929 32369450 PMC7308058

[B3] Altman B. J. Stine Z. E. Dang C. V. (2016). From krebs to clinic: Glutamine metabolism to cancer therapy. Nat. Rev. Cancer 16, 619–634. 10.1038/nrc.2016.71 27492215 PMC5484415

[B4] Andrejeva G. Rathmell J. C. (2017). Similarities and distinctions of cancer and immune metabolism in inflammation and tumors. Cell Metab. 26, 49–70. 10.1016/j.cmet.2017.06.004 28683294 PMC5555084

[B5] Angelini G. Gardella S. Ardy M. Ciriolo M. R. Filomeni G. Di Trapani G. (2002). Antigen-presenting dendritic cells provide the reducing extracellular microenvironment required for T lymphocyte activation. Proc. Natl. Acad. Sci. U. S. A. 99, 1491–1496. 10.1073/pnas.022630299 11792859 PMC122218

[B6] Araki K. Turner A. P. Shaffer V. O. Gangappa S. Keller S. A. Bachmann M. F. (2009). mTOR regulates memory CD8 T-cell differentiation. Nature 460, 108–112. 10.1038/nature08155 19543266 PMC2710807

[B7] Arensman M. D. Yang X. S. Leahy D. M. Toral-Barza L. Mileski M. Rosfjord E. C. (2019). Cystine–glutamate antiporter xCT deficiency suppresses tumor growth while preserving antitumor immunity. Proc. Natl. Acad. Sci. U. S. A. 116, 9533–9542. 10.1073/pnas.1814932116 31019077 PMC6511047

[B8] Bian Y. Li W. Kremer D. M. Sajjakulnukit P. Li S. Crespo J. (2020). Cancer SLC43A2 alters T cell methionine metabolism and histone methylation. Nature 585, 277–282. 10.1038/s41586-020-2682-1 32879489 PMC7486248

[B9] Boudreau A. Purkey H. E. Hitz A. Robarge K. Peterson D. Labadie S. (2016). Metabolic plasticity underpins innate and acquired resistance to LDHA inhibition. Nat. Chem. Biol. 12, 779–786. 10.1038/nchembio.2143 27479743

[B10] Brand A. Singer K. Koehl G. E. Kolitzus M. Schoenhammer G. Thiel A. (2016). LDHA-associated lactic acid production blunts tumor immunosurveillance by T and NK cells. Cell Metab. 24, 657–671. 10.1016/j.cmet.2016.08.011 27641098

[B11] Carr E. L. Kelman A. Wu G. S. Gopaul R. Senkevitch E. Aghvanyan A. (2010). Glutamine uptake and metabolism are coordinately regulated by ERK/MAPK during T lymphocyte activation. J. Immunol. 185, 1037–1044. 10.4049/jimmunol.0903586 20554958 PMC2897897

[B12] Cascone T. McKenzie J. A. Mbofung R. M. Punt S. Wang Z. Xu C. (2018). Increased tumor glycolysis characterizes immune resistance to adoptive T cell therapy. Cell Metab. 27, 977–987. 10.1016/j.cmet.2018.02.024 29628419 PMC5932208

[B13] Chamoto K. Chowdhury P. S. Kumar A. Sonomura K. Matsuda F. Fagarasan S. (2017). Mitochondrial activation chemicals synergize with surface receptor PD-1 blockade for T cell-dependent antitumor activity. Proc. Natl. Acad. Sci. U. S. A. 114, E761–E770. 10.1073/pnas.1620433114 28096382 PMC5293087

[B14] Chang C.-H. Qiu J. O’Sullivan D. Buck M. D. Noguchi T. Curtis J. D. (2015). Metabolic competition in the tumor microenvironment is a driver of cancer progression. Cell 162, 1229–1241. 10.1016/j.cell.2015.08.016 26321679 PMC4864363

[B15] Chaoul N. Fayolle C. Desrues B. Oberkampf M. Tang A. Ladant D. (2015). Rapamycin impairs antitumor CD8+ T-cell responses and vaccine-induced tumor eradication. Cancer Res. 75, 3279–3291. 10.1158/0008-5472.CAN-15-0454 26122844

[B16] Chen W. Jia W. Wu C. Chen L. Sun K. Wang J. (2021). The neurogenic compound P7C3 regulates the aerobic glycolysis by targeting phosphoglycerate kinase 1 in glioma. Front. Oncol. 11, 644492. 10.3389/fonc.2021.644492 34221965 PMC8252887

[B17] Cheng T. Sudderth J. Yang C. Mullen A. R. Jin E. S. Matés J. M. (2011). Pyruvate carboxylase is required for glutamine-independent growth of tumor cells. Proc. Natl. Acad. Sci. U. S. A. 108, 8674–8679. 10.1073/pnas.1016627108 21555572 PMC3102381

[B18] Chernick R. J. Martasek P. Levere R. D. Margreiter R. Abraham N. G. (1989). Sensitivity of human tissue heme oxygenase to a new synthetic metalloporphyrin. Hepatol. Balt. Md 10, 365–369. 10.1002/hep.1840100320 2759552

[B19] Chong J. Wishart D. S. Xia J. (2019). Using MetaboAnalyst 4.0 for comprehensive and integrative metabolomics data analysis. Curr. Protoc. Bioinforma. 68, e86. 10.1002/cpbi.86 31756036

[B20] Crump N. T. Hadjinicolaou A. V. Xia M. Walsby-Tickle J. Gileadi U. Chen J.-L. (2021). Chromatin accessibility governs the differential response of cancer and T cells to arginine starvation. Cell Rep. 35, 109101. 10.1016/j.celrep.2021.109101 33979616 PMC8131582

[B21] Curthoys N. P. Watford M. (1995). Regulation of glutaminase activity and glutamine metabolism. Annu. Rev. Nutr. 15, 133–159. 10.1146/annurev.nu.15.070195.001025 8527215

[B22] Dadi S. Chhangawala S. Whitlock B. M. Franklin R. A. Luo C. T. Oh S. A. (2016). Cancer immunosurveillance by tissue-resident innate lymphoid cells and innate-like T cells. Cell 164, 365–377. 10.1016/j.cell.2016.01.002 26806130 PMC4733424

[B23] Dai X. Bu X. Gao Y. Guo J. Hu J. Jiang C. (2021). Energy status dictates PD-L1 protein abundance and anti-tumor immunity to enable checkpoint blockade. Mol. Cell 81, 2317–2331.e6. 10.1016/j.molcel.2021.03.037 33909988 PMC8178223

[B24] Davidson S. M. Jonas O. Keibler M. A. Hou H. W. Luengo A. Mayers J. R. (2017). Direct evidence for cancer-cell-autonomous extracellular protein catabolism in pancreatic tumors. Nat. Med. 23, 235–241. 10.1038/nm.4256 28024083 PMC5407288

[B25] Davidson S. M. Papagiannakopoulos T. Olenchock B. A. Heyman J. E. Keibler M. A. Luengo A. (2016). Environment impacts the metabolic dependencies of ras-driven non-small cell lung cancer. Cell Metab. 23, 517–528. 10.1016/j.cmet.2016.01.007 26853747 PMC4785096

[B26] Deaglio S. Dwyer K. M. Gao W. Friedman D. Usheva A. Erat A. (2007). Adenosine generation catalyzed by CD39 and CD73 expressed on regulatory T cells mediates immune suppression. J. Exp. Med. 204, 1257–1265. 10.1084/jem.20062512 17502665 PMC2118603

[B27] Egeblad M. Nakasone E. S. Werb Z. (2010). Tumors as organs: Complex tissues that interface with the entire organism. Dev. Cell 18, 884–901. 10.1016/j.devcel.2010.05.012 20627072 PMC2905377

[B28] Feng Q. Liu Z. Yu X. Huang T. Chen J. Wang J. (2022). Lactate increases stemness of CD8 + T cells to augment anti-tumor immunity. Nat. Commun. 13 (1), 4981. 10.1038/s41467-022-32521-8 36068198 PMC9448806

[B29] Fischer K. Hoffmann P. Voelkl S. Meidenbauer N. Ammer J. Edinger M. (2007). Inhibitory effect of tumor cell-derived lactic acid on human T cells. Blood 109, 3812–3819. 10.1182/blood-2006-07-035972 17255361

[B30] Fridman W. H. Zitvogel L. Sautès-Fridman C. Kroemer G. (2017). The immune contexture in cancer prognosis and treatment. Nat. Rev. Clin. Oncol. 14, 717–734. 10.1038/nrclinonc.2017.101 28741618

[B31] Garg S. K. Yan Z. Vitvitsky V. Banerjee R. (2011). Differential dependence on cysteine from transsulfuration versus transport during T cell activation. Antioxid. Redox Signal. 15, 39–47. 10.1089/ars.2010.3496 20673163 PMC3110100

[B32] Geiger R. Rieckmann J. C. Wolf T. Basso C. Feng Y. Fuhrer T. (2016). L-arginine modulates T cell metabolism and enhances survival and anti-tumor activity. Cell 167, 829–842. 10.1016/j.cell.2016.09.031 27745970 PMC5075284

[B33] Gmünder H. Eck H. P. Benninghoff B. Roth S. Dröge W. (1990). Macrophages regulate intracellular glutathione levels of lymphocytes. Evidence for an immunoregulatory role of cysteine. Cell. Immunol. 129, 32–46. 10.1016/0008-8749(90)90184-s 2364441

[B34] Gout P. W. Buckley A. R. Simms C. R. Bruchovsky N. (2001). Sulfasalazine, a potent suppressor of lymphoma growth by inhibition of the x(c)- cystine transporter: A new action for an old drug. Leukemia 15, 1633–1640. 10.1038/sj.leu.2402238 11587223

[B35] Gout P. W. Simms C. R. Robertson M. C. (2003). *In vitro* studies on the lymphoma growth-inhibitory activity of sulfasalazine. Anticancer. Drugs 14, 21–29. 10.1097/00001813-200301000-00004 12544255

[B36] Griffith O. W. Meister A. (1979). Potent and specific inhibition of glutathione synthesis by buthionine sulfoximine (S-n-butyl homocysteine sulfoximine). J. Biol. Chem. 254, 7558–7560. 10.1016/s0021-9258(18)35980-5 38242

[B37] Guy C. T. Cardiff R. D. Muller W. J. (1992). Induction of mammary tumors by expression of polyomavirus middle T oncogene: A transgenic mouse model for metastatic disease. Mol. Cell. Biol. 12, 954–961. 10.1128/mcb.12.3.954 1312220 PMC369527

[B38] Halbrook C. J. Pontious C. Kovalenko I. Lapienyte L. Dreyer S. Lee H.-J. (2019). Macrophage-released pyrimidines inhibit gemcitabine therapy in pancreatic cancer. Cell Metab. 29, 1390–1399. e6. 10.1016/j.cmet.2019.02.001 30827862 PMC6602533

[B39] Hardwicke M. A. Rendina A. R. Williams S. P. Moore M. L. Wang L. Krueger J. A. (2014). A human fatty acid synthase inhibitor binds β-ketoacyl reductase in the keto-substrate site. Nat. Chem. Biol. 10, 774–779. 10.1038/nchembio.1603 25086508

[B40] Has-71291 R. (2022). Reactome.org 81. Available at: https://reactome.org/download-data/ .

[B41] Henrich F. C. Singer K. Poller K. Bernhardt L. Strobl C. D. Limm K. (2016). Suppressive effects of tumor cell-derived 5′-deoxy-5′-methylthioadenosine on human T cells. OncoImmunology 5, e1184802. 10.1080/2162402X.2016.1184802 27622058 PMC5007975

[B42] Hossain F. Al-Khami A. A. Wyczechowska D. Hernandez C. Zheng L. Reiss K. (2015). Inhibition of fatty acid oxidation modulates immunosuppressive functions of myeloid-derived suppressor cells and enhances cancer therapies. Cancer Immunol. Res. 3, 1236–1247. 10.1158/2326-6066.CIR-15-0036 26025381 PMC4636942

[B43] Hung M. H. Lee J. S. Ma C. Diggs L. P. Heinrich S. Chang C. W. (2021). Tumor methionine metabolism drives T-cell exhaustion in hepatocellular carcinoma. Nat. Commun. 12, 1455. 10.1038/s41467-021-21804-1 33674593 PMC7935900

[B44] Investor Agios (2021). Agios provides update on PKR program | agios pharmaceuticals, Inc. Available at: https://investor.agios.com/news-releases/news-release-details/agios-provides-update-pkr-program (accessed 7.7.21).

[B45] Jaworski F. M. Gentilini L. D. Gueron G. Meiss R. P. Ortiz E. G. Berguer P. M. (2017). *In vivo* hemin conditioning targets the vascular and immunologic compartments and restrains prostate tumor development. Clin. Cancer Res. 23, 5135–5148. 10.1158/1078-0432.CCR-17-0112 28512172

[B46] Jonas O. Landry H. M. Fuller J. E. Santini J. T. Baselga J. Tepper R. I. (2015). An implantable microdevice to perform high-throughput *in vivo* drug sensitivity testing in tumors. Sci. Transl. Med. 7, 284ra57. 10.1126/scitranslmed.3010564 PMC4825177 25904741

[B47] Kiorpes T. C. Hoerr D. Ho W. Weaner L. E. Inman M. G. Tutwiler G. F. (1984). Identification of 2-tetradecylglycidyl coenzyme A as the active form of methyl 2-tetradecylglycidate (methyl palmoxirate) and its characterization as an irreversible, active site-directed inhibitor of carnitine palmitoyltransferase A in isolated rat liver mitochondria. J. Biol. Chem. 259, 9750–9755. 10.1016/s0021-9258(17)42763-3 6547720

[B48] Labadie B. W. Bao R. Luke J. J. (2019). Reimagining Ido pathway inhibition in cancer immunotherapy via downstream focus on the tryptophan-kynurenine-aryl hydrocarbon Axis. Clin. Cancer Res. Off. J. Am. Assoc. Cancer Res. 25, 1462–1471. 10.1158/1078-0432.CCR-18-2882 PMC6397695 30377198

[B49] Lampropoulou V. Sergushichev A. Bambouskova M. Nair S. Vincent E. E. Loginicheva E. (2016). Itaconate links inhibition of succinate dehydrogenase with macrophage metabolic remodeling and regulation of inflammation. Cell Metab. 24, 158–166. 10.1016/j.cmet.2016.06.004 27374498 PMC5108454

[B50] Lancien M. Gueno L. Salle S. Merieau E. Beriou G. Nguyen T. H. (2021). Cystathionine-gamma-lyase overexpression in T cells enhances antitumor effect independently of cysteine autonomy. Cancer Sci. 112, 1723–1734. 10.1111/cas.14862 33609296 PMC8088958

[B51] Laplante M. Sabatini D. M. (2012). mTOR signaling in growth control and disease. Cell 149, 274–293. 10.1016/j.cell.2012.03.017 22500797 PMC3331679

[B52] Leone R. D. Powell J. D. (2020). Metabolism of immune cells in cancer. Nat. Rev. Cancer 20, 516–531. 10.1038/s41568-020-0273-y 32632251 PMC8041116

[B53] Leone R. D. Sun I.-M. Oh M.-H. Sun I.-H. Wen J. Englert J. (2018). Inhibition of the adenosine A2a receptor modulates expression of T cell coinhibitory receptors and improves effector function for enhanced checkpoint blockade and ACT in murine cancer models. Cancer Immunol. Immunother. CII 67, 1271–1284. 10.1007/s00262-018-2186-0 29923026 PMC11028354

[B54] Leone R. D. Zhao L. Englert J. M. Sun I.-M. Oh M.-H. Sun I.-H. (2019). Glutamine blockade induces divergent metabolic programs to overcome tumor immune evasion. Science 366, 1013–1021. 10.1126/science.aav2588 31699883 PMC7023461

[B55] Levring T. B. Hansen A. K. Nielsen B. L. Kongsbak M. von Essen M. R. Woetmann A. (2012). Activated human CD4+ T cells express transporters for both cysteine and cystine. Sci. Rep. 2, 266. 10.1038/srep00266 22355778 PMC3278673

[B56] Li Q. Rao R. R. Araki K. Pollizzi K. Odunsi K. Powell J. D. (2011). A central role for mTOR kinase in homeostatic proliferation induced CD8+ T cell memory and tumor immunity. Immunity 34, 541–553. 10.1016/j.immuni.2011.04.006 21511183 PMC3083826

[B57] Lin E. Y. Jones J. G. Li P. Zhu L. Whitney K. D. Muller W. J. (2003). Progression to malignancy in the polyoma middle T oncoprotein mouse breast cancer model provides a reliable model for human diseases. Am. J. Pathol. 163, 2113–2126. 10.1016/S0002-9440(10)63568-7 14578209 PMC1892434

[B58] Liu M. Wang X. Wang L. Ma X. Gong Z. Zhang S. (2018). Targeting the Ido1 pathway in cancer: From bench to bedside. J. Hematol. Oncol.J Hematol. Oncol. 11, 100. 10.1186/s13045-018-0644-y 30068361 PMC6090955

[B59] Liu N. Zhang J. Yin M. Liu H. Zhang X. Li J. (2021). Inhibition of xCT suppresses the efficacy of anti-PD-1/L1 melanoma treatment through exosomal PD-L1-induced macrophage M2 polarization. Mol. Ther. J. Am. Soc. Gene Ther. S1525-0016 (21), 2321–2334. 10.1016/j.ymthe.2021.03.013 PMC8261162 33744468

[B60] Liu Y. Liang X. Dong W. Fang Y. Lv J. Zhang T. (2018). Tumor-repopulating cells induce PD-1 expression in CD8+ T cells by transferring kynurenine and AhR activation. Cancer Cell 33, 480–494. 10.1016/j.ccell.2018.02.005 29533786

[B61] Ma E. H. Verway M. J. Johnson R. M. Roy D. G. Steadman M. Hayes S. (2019). Metabolic profiling using stable isotope tracing reveals distinct patterns of glucose utilization by physiologically activated CD8+ T cells. Immunity 51, 856–870. 10.1016/j.immuni.2019.09.003 31747582

[B62] Muliaditan T. Opzoomer J. W. Caron J. Okesola M. Kosti P. Lall S. (2018). Repurposing tin mesoporphyrin as an immune checkpoint inhibitor shows therapeutic efficacy in preclinical models of cancer. Clin. Cancer Res. Off. J. Am. Assoc. Cancer Res. 24, 1617–1628. 10.1158/1078-0432.CCR-17-2587 PMC5889101 29339440

[B63] Munn D. H. Shafizadeh E. Attwood J. T. Bondarev I. Pashine A. Mellor A. L. (1999). Inhibition of T cell proliferation by macrophage tryptophan catabolism. J. Exp. Med. 189, 1363–1372. 10.1084/jem.189.9.1363 10224276 PMC2193062

[B64] Nakaya M. Xiao Y. Zhou X. Chang J.-H. Chang M. Cheng X. (2014). Inflammatory T cell responses rely on amino acid transporter ASCT2 facilitation of glutamine uptake and mTORC1 kinase activation. Immunity 40, 692–705. 10.1016/j.immuni.2014.04.007 24792914 PMC4074507

[B65] O’Connor R. S. Guo L. Ghassemi S. Snyder N. W. Worth A. J. Weng L. (2018). The CPT1a inhibitor, etomoxir induces severe oxidative stress at commonly used concentrations. Sci. Rep. 8, 6289. 10.1038/s41598-018-24676-6 29674640 PMC5908836

[B66] Ohta A. Gorelik E. Prasad S. J. Ronchese F. Lukashev D. Wong M. K. K. (2006). A2A adenosine receptor protects tumors from antitumor T cells. Proc. Natl. Acad. Sci. 103, 13132–13137. 10.1073/pnas.0605251103 16916931 PMC1559765

[B67] Pacella I. Procaccini C. Focaccetti C. Miacci S. Timperi E. Faicchia D. (2018). Fatty acid metabolism complements glycolysis in the selective regulatory T cell expansion during tumor growth. Proc. Natl. Acad. Sci. 115, E6546–E6555. 10.1073/pnas.1720113115 29941600 PMC6048537

[B68] Pang Z. Chong J. Zhou G. de Lima Morais D. A. Chang L. Barrette M. (2021). MetaboAnalyst 5.0: Narrowing the gap between raw spectra and functional insights. Nucleic Acids Res. 49, W388–W396. 10.1093/nar/gkab382 34019663 PMC8265181

[B69] Patel C. H. Leone R. D. Horton M. R. Powell J. D. (2019). Targeting metabolism to regulate immune responses in autoimmunity and cancer. Nat. Rev. Drug Discov. 18, 669–688. 10.1038/s41573-019-0032-5 31363227

[B70] Pavlova N. N. Thompson C. B. (2016). The emerging hallmarks of cancer metabolism. Cell Metab. 23, 27–47. 10.1016/j.cmet.2015.12.006 26771115 PMC4715268

[B71] Pieper A. A. Xie S. Capota E. Estill S. J. Zhong J. Long J. M. (2010). Discovery of a proneurogenic, neuroprotective chemical. Neuroprotective Chem. Cell 142, 39–51. 10.1016/j.cell.2010.06.018 PMC2930815 20603013

[B72] Pollizzi K. N. Patel C. H. Sun I.-H. Oh M.-H. Waickman A. T. Wen J. (2015). mTORC1 and mTORC2 selectively regulate CD8⁺ T cell differentiation. J. Clin. Invest. 125, 2090–2108. 10.1172/JCI77746 25893604 PMC4463194

[B73] Rad Pour S. Morikawa H. Kiani N. A. Yang M. Azimi A. Shafi G. (2019). Exhaustion of CD4+ T-cells mediated by the kynurenine pathway in melanoma. Sci. Rep. 9, 12150. 10.1038/s41598-019-48635-x 31434983 PMC6704156

[B74] Ren W. Liu G. Yin J. Tan B. Wu G. Bazer F. W. (2017). Amino-acid transporters in T-cell activation and differentiation. Cell Death Dis. 8, e2655. 10.1038/cddis.2016.222 28252650 PMC5386510

[B75] Robinson M. M. McBryant S. J. Tsukamoto T. Rojas C. Ferraris D. V. Hamilton S. K. (2007). Novel mechanism of inhibition of rat kidney-type glutaminase by bis-2-(5-phenylacetamido-1, 2, 4-thiadiazol-2-yl)ethyl sulfide (BPTES). Biochem. J. 406, 407–414. 10.1042/BJ20070039 17581113 PMC2049044

[B76] Rodriguez P. C. Quiceno D. G. Ochoa A. C. (2007). L-arginine availability regulates T-lymphocyte cell-cycle progression. Blood 109, 1568–1573. 10.1182/blood-2006-06-031856 17023580 PMC1794048

[B77] Routy J.-P. Routy B. Graziani G. M. Mehraj V. (2016). The kynurenine pathway is a double-edged sword in immune-privileged sites and in cancer: Implications for immunotherapy. Int. J. Tryptophan Res. IJTR 9, 67–77. 10.4137/IJTR.S38355 27773992 PMC5063567

[B78] Roux C. Jafari S. M. Shinde R. Duncan G. Cescon D. W. Silvester J. (2019). Reactive oxygen species modulate macrophage immunosuppressive phenotype through the up-regulation of PD-L1. Proc. Natl. Acad. Sci. 116, 4326–4335. 10.1073/pnas.1819473116 30770442 PMC6410837

[B79] Sabatini D. M. Pierchala B. A. Barrow R. K. Schell M. J. Snyder S. H. (1995). The rapamycin and FKBP12 target (RAFT) displays phosphatidylinositol 4-kinase activity. J. Biol. Chem. 270, 20875–20878. 10.1074/jbc.270.36.20875 7673106

[B80] Singha P. K. Mäklin K. Vihavainen T. Laitinen T. Nevalainen T. J. Patil M. R. (2020). Evaluation of FASN inhibitors by a versatile toolkit reveals differences in pharmacology between human and rodent FASN preparations and in antiproliferative efficacy *in vitro* vs. *in situ* in human cancer cells. Eur. J. Pharm. Sci. Off. J. Eur. Fed. Pharm. Sci. 149, 105321. 10.1016/j.ejps.2020.105321 32275951

[B81] Siska P. J. Kim B. Ji X. Hoeksema M. D. Massion P. P. Beckermann K. E. (2016). Fluorescence-based measurement of cystine uptake through xCT shows requirement for ROS detoxification in activated lymphocytes. J. Immunol. Methods 438, 51–58. 10.1016/j.jim.2016.08.013 27594594 PMC5065394

[B82] Srivastava M. K. Sinha P. Clements V. K. Rodriguez P. Ostrand-Rosenberg S. (2010). Myeloid-Derived suppressor cells inhibit T-cell activation by depleting cystine and cysteine. Cancer Res. 70, 68–77. 10.1158/0008-5472.CAN-09-2587 20028852 PMC2805057

[B83] Strobl C. D. Schaffer S. Haug T. Völkl S. Peter K. Singer K. (2020). Selective PRMT5 inhibitors suppress human CD8+ T cells by upregulation of p53 and impairment of the AKT pathway similar to the tumor metabolite MTA. Mol. Cancer Ther. 19, 409–419. 10.1158/1535-7163.MCT-19-0189 31712395

[B84] Thangavelu K. Pan C. Q. Karlberg T. Balaji G. Uttamchandani M. Suresh V. (2012). Structural basis for the allosteric inhibitory mechanism of human kidney-type glutaminase (KGA) and its regulation by Raf-Mek-Erk signaling in cancer cell metabolism. Proc. Natl. Acad. Sci. 109, 7705–7710. 10.1073/pnas.1116573109 22538822 PMC3356676

[B85] van Gastel N. Spinelli J. B. Sharda A. Schajnovitz A. Baryawno N. Rhee C. (2020). Induction of a timed metabolic collapse to overcome cancer chemoresistance. Cell Metab. 32, 391–403. e6. 10.1016/j.cmet.2020.07.009 32763164 PMC8397232

[B86] Waldman A. D. Fritz J. M. Lenardo M. J. (2020). A guide to cancer immunotherapy: from T cell basic science to clinical practice. Nat. Rev. Immunol. 20, 651–668. 10.1038/s41577-020-0306-5 32433532 PMC7238960

[B87] Wang G. Han T. Nijhawan D. Theodoropoulos P. Naidoo J. Yadavalli S. (2014). P7C3 neuroprotective chemicals function by activating the rate-limiting enzyme in NAD salvage. Cell 158, 1324–1334. 10.1016/j.cell.2014.07.040 25215490 PMC4163014

[B88] Wang R. Dillon C. P. Shi L. Z. Milasta S. Carter R. Finkelstein D. (2011). The transcription factor Myc controls metabolic reprogramming upon T lymphocyte activation. Immunity 35, 871–882. 10.1016/j.immuni.2011.09.021 22195744 PMC3248798

[B89] Warburg O. (1956). On the origin of cancer cells. Science 123, 309–314. 10.1126/science.123.3191.309 13298683

[B90] Weiss J. M. Davies L. C. Karwan M. Ileva L. Ozaki M. K. Cheng R. Y. (2018). Itaconic acid mediates crosstalk between macrophage metabolism and peritoneal tumors. J. Clin. Invest. 128, 3794–3805. 10.1172/JCI99169 29920191 PMC6118601

[B91] Wenes M. Jaccard A. Wyss T. Maldonado-Pérez N. Teoh S. T. Lepez A. (2022). The mitochondrial pyruvate carrier regulates memory T cell differentiation and antitumor function. Cell Metab. 34 (5), 731–746. 10.1016/j.cmet.2022.03.013 35452600 PMC9116152

[B92] Xia J. Psychogios N. Young N. Wishart D. S. (2009). MetaboAnalyst: A web server for metabolomic data analysis and interpretation. Nucleic Acids Res. 37, W652–W660. 10.1093/nar/gkp356 19429898 PMC2703878

[B93] Ždralević M. Brand A. Di Ianni L. Dettmer K. Reinders J. Singer K. (2018). Double genetic disruption of lactate dehydrogenases A and B is required to ablate the “Warburg effect” restricting tumor growth to oxidative metabolism. J. Biol. Chem. 293, 15947–15961. 10.1074/jbc.RA118.004180 30158244 PMC6187639

