# Supplementary material for: High-throughput in situ perturbation of metabolite levels in the tumor micro-environment reveals favorable metabolic condition for increased fitness of infiltrated T-cells
Source: Front Cell Dev Biol. 2022 Dec 22;10:1032360. doi: 10.3389/fcell.2022.1032360 (PMC9815512; doi:10.3389/fcell.2022.1032360)
Supplement: Supplementary file 1 [file DataSheet1.PDF]

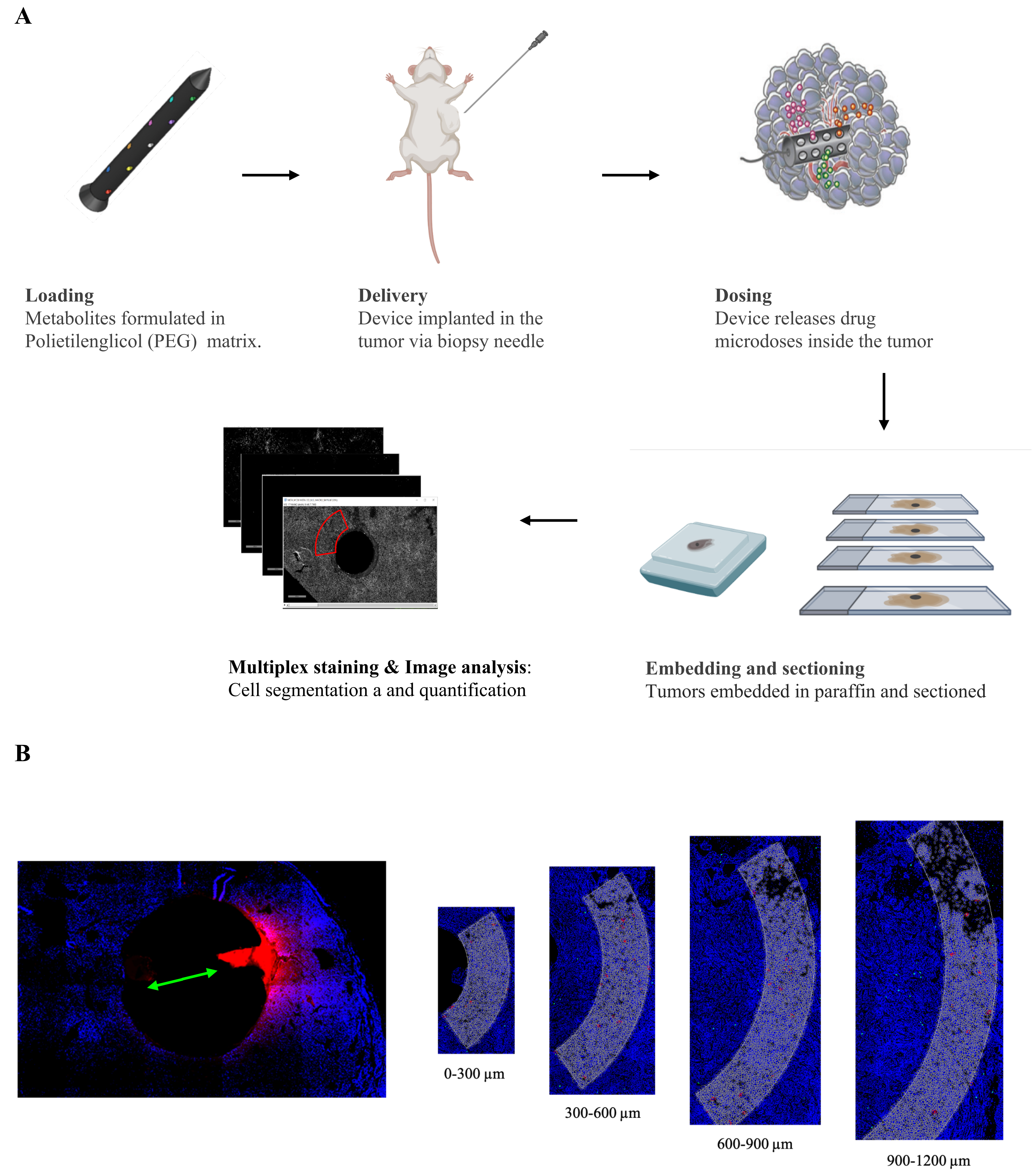
A
Loading
Metabolites formulated in
Polietilenglicol (PEG) matrix.
Delivery
Device implanted in the
tumor via biopsy needle
Dosing
Device releases drug
microdoses inside the tumor
Multiplex staining & Image analysis:
Cell segmentation a and quantification
Embedding and sectioning
Tumors embedded in paraffin and sectioned
B
0-300 µm
300-600 µm
600-900 µm
900-1200 µm

Fig.1

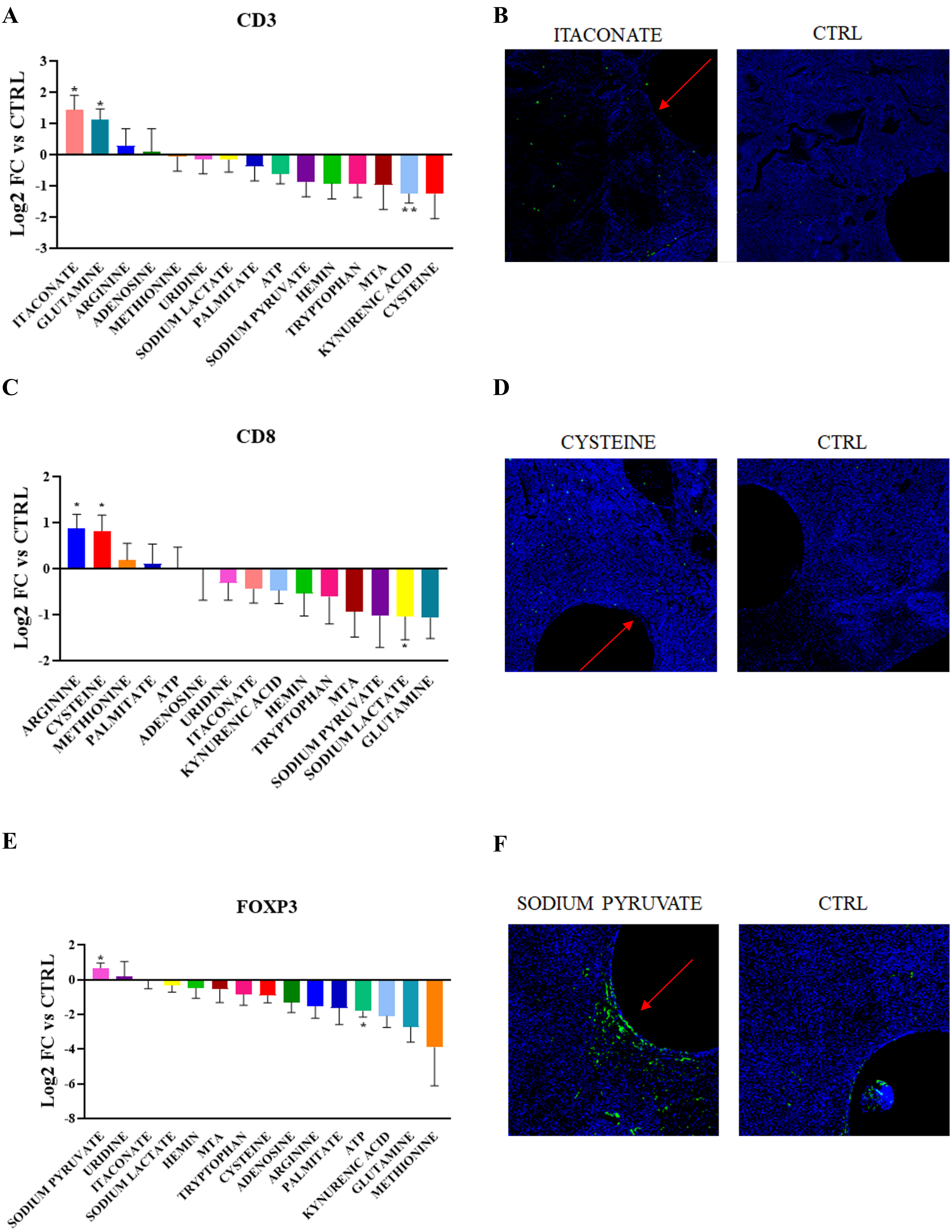
A
CD3
Log2 FC vs CTRL
3
2
1
0
-1
-2
-3
*
*
**
ITACONATE
GLUTAMINE
ARGININE
ADENOSINE
METHIONINE
URIDINE
SODIUM LACTATE
PALMITATE
ATP
SODIUM PYRUVATE
HEMIN
TRYPTOPHAN
MTA
KYNURENIC ACID
CYSTEINE
B
ITACONATE
CTRL
C
CD8
Log2 FC vs CTRL
2
1
0
-1
-2
*
*
*
ARGININE
CYSTEINE
METHIONINE
PALMITATE
ATP
ADENOSINE
URIDINE
ITACONATE
KYNURENIC ACID
HEMIN
TRYPTOPHAN
MTA
SODIUM PYRUVATE
SODIUM LACTATE
GLUTAMINE
D
CYSTEINE
CTRL
E
FOXP3
Log2 FC vs CTRL
2
0
-2
-4
-6
-8
*
*
SODIUM PYRUVATE
URIDINE
ITACONATE
SODIUM LACTATE
HEMIN
MTA
TRYPTOPHAN
CYSTEINE
ADENOSINE
ARGININE
PALMITATE
ATP
KYNURENIC ACID
GLUTAMINE
METHIONINE
F
SODIUM PYRUVATE
CTRL

Fig.2

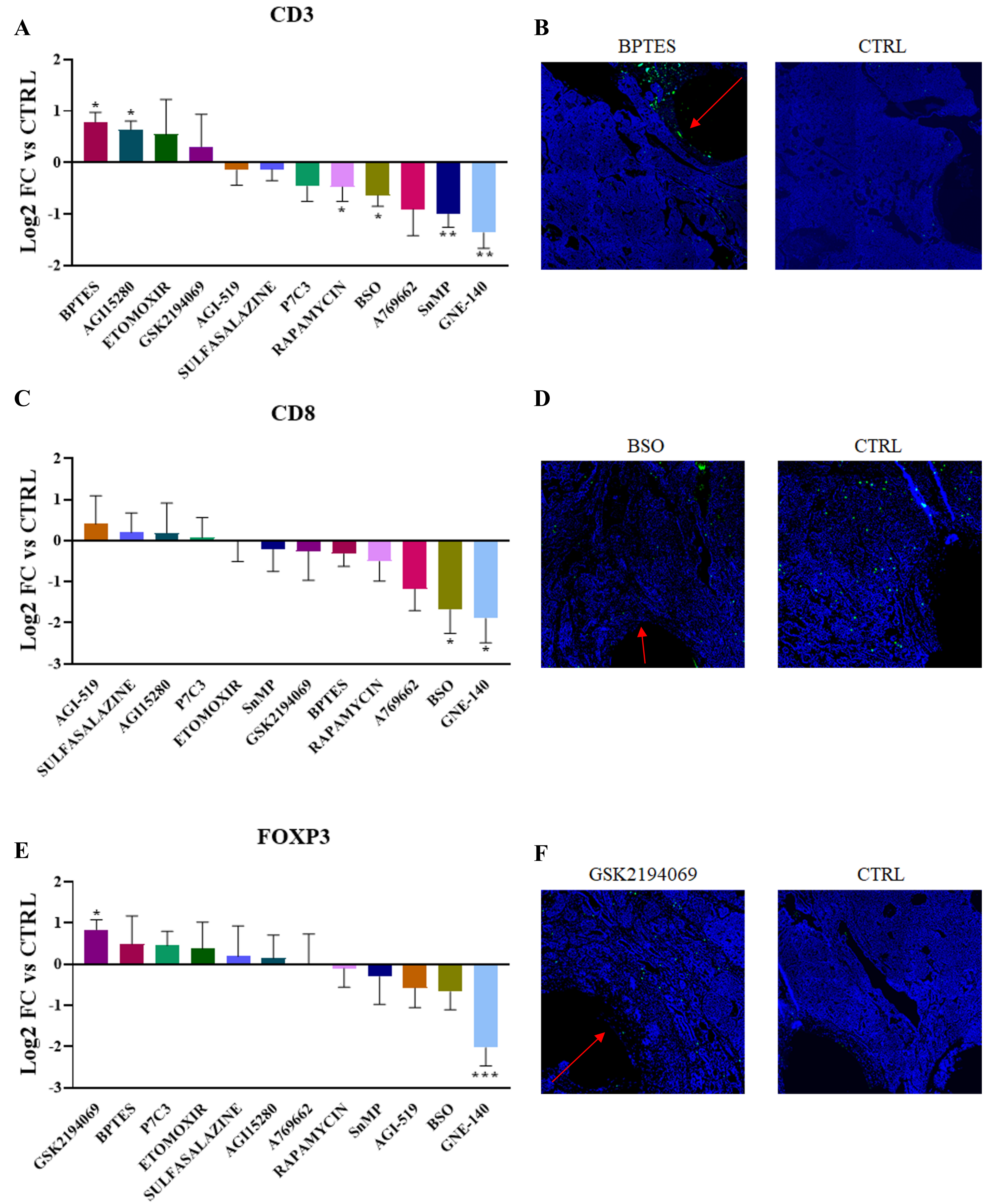
A
CD3
Log2 FC vs CTRL
2
1
0
-1
-2
*
*
*
*
**
**
BPTES
AGI5280
ETOMOXIR
GSK2194069
AGI-519
SULFASALAZINE
P7C3
RAPAMYCIN
BSO
A769662
SnMP
GNE-140
B
BPTES
CTRL
C
CD8
Log2 FC vs CTRL
2
1
0
-1
-2
-3
*
*
AGI-519
SULFASALAZINE
AGI5280
P7C3
ETOMOXIR
SnMP
GSK2194069
BPTES
RAPAMYCIN
A769662
BSO
GNE-140
D
BSO
CTRL
E
FOXP3
Log2 FC vs CTRL
2
1
0
-1
-2
-3
*
***
GSK2194069
BPTES
P7C3
ETOMOXIR
SULFASALAZINE
AGI5280
A769662
RAPAMYCIN
SnMP
AGI-519
BSO
GNE-140
F
GSK2194069
CTRL

Fig.3

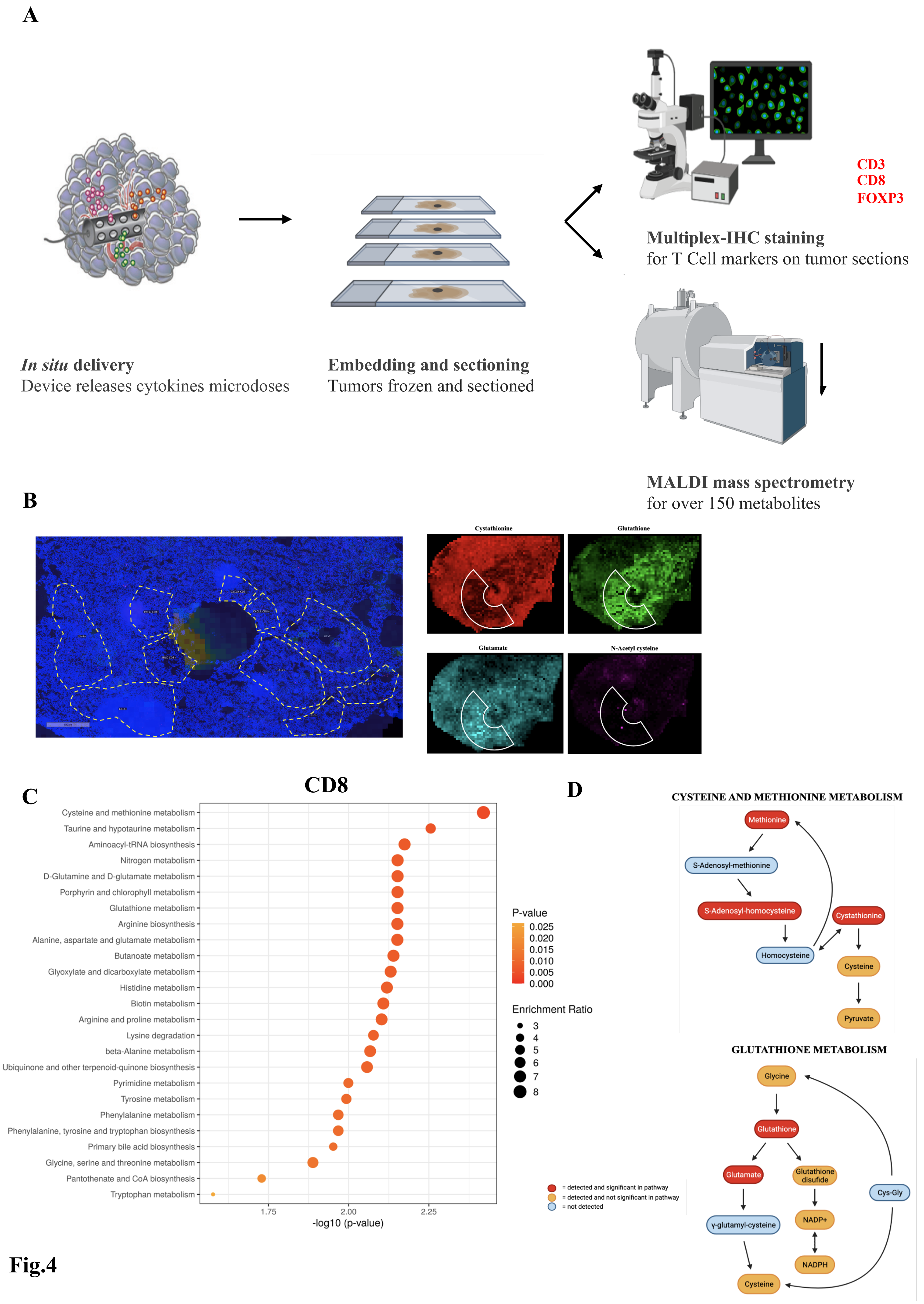
A
In situ delivery
Device releases cytokines microdoses
Embedding and sectioning
Tumors frozen and sectioned
CD3
CD8
FOXP3
Multiplex-IHC staining
for T Cell markers on tumor sections
MALDI mass spectrometry
for over 150 metabolites
B
Cystathionine
Glutathione
Glutamate
N-Acetyl cysteine
C
CD8
Cysteine and methionine metabolism
Taurine and hypotaurine metabolism
Aminoacyl-tRNA biosynthesis
Nitrogen metabolism
D-Glutamine and D-glutamate metabolism
Porphyrin and chlorophyll metabolism
Glutathione metabolism
Arginine biosynthesis
Alanine, aspartate and glutamate metabolism
Butanoate metabolism
Glyoxylate and dicarboxylate metabolism
Histidine metabolism
Biotin metabolism
Arginine and proline metabolism
Lysine degradation
beta-Alanine metabolism
Ubiquinone and other terpenoid-quinone biosynthesis
Pyrimidine metabolism
Tyrosine metabolism
Phenylalanine metabolism
Phenylalanine, tyrosine and tryptophan biosynthesis
Primary bile acid biosynthesis
Glycine, serine and threonine metabolism
Pantothenate and CoA biosynthesis
Tryptophan metabolism
1.75
2.00
2.25
-log10 (p-value)
P-value
0.025
0.020
0.015
0.010
0.005
0.000
Enrichment Ratio
3
4
5
6
7
8
D
CYSTEINE AND METHIONINE METABOLISM
Methionine
S-Adenosyl-methionine
S-Adenosyl-homocysteine
Cystathionine
Homocysteine
Cysteine
Pyruvate
GLUTATHIONE METABOLISM
Glycine
Glutathione
Glutamate
Glutathione disufide
Cys-Gly
γ-glutamyl-cysteine
NADP+
NADPH
Cysteine
= detected and significant in pathway
= detected and not significant in pathway
= not detected

Fig.4

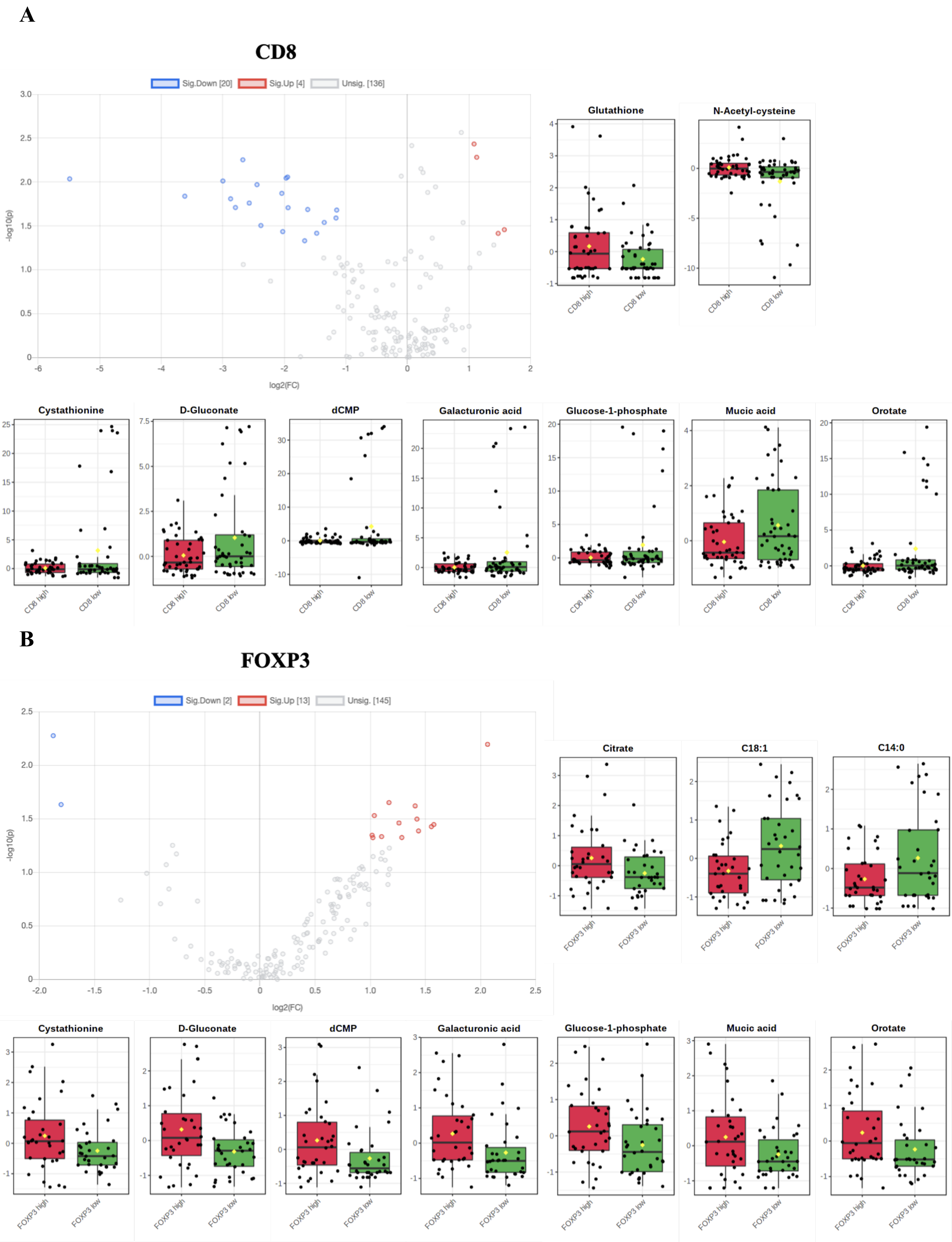
A
CD8
Sig.Down [20]
Sig.Up [4]
Unsig. [136]
-log10(p)
log2(FC)
Glutathione
N-Acetyl-cysteine
Cystathionine
D-Gluconate
dCMP
Galacturonic acid
Glucose-1-phosphate
Mucic acid
Orotate
CD8 high
CD8 low
B
FOXP3
Sig.Down [2]
Sig.Up [13]
Unsig. [145]
-log10(p)
log2(FC)
Citrate
C18:1
C14:0
Cystathionine
D-Gluconate
dCMP
Galacturonic acid
Glucose-1-phosphate
Mucic acid
Orotate
FOXP3 high
FOXP3 low

Fig.5

A

CD8

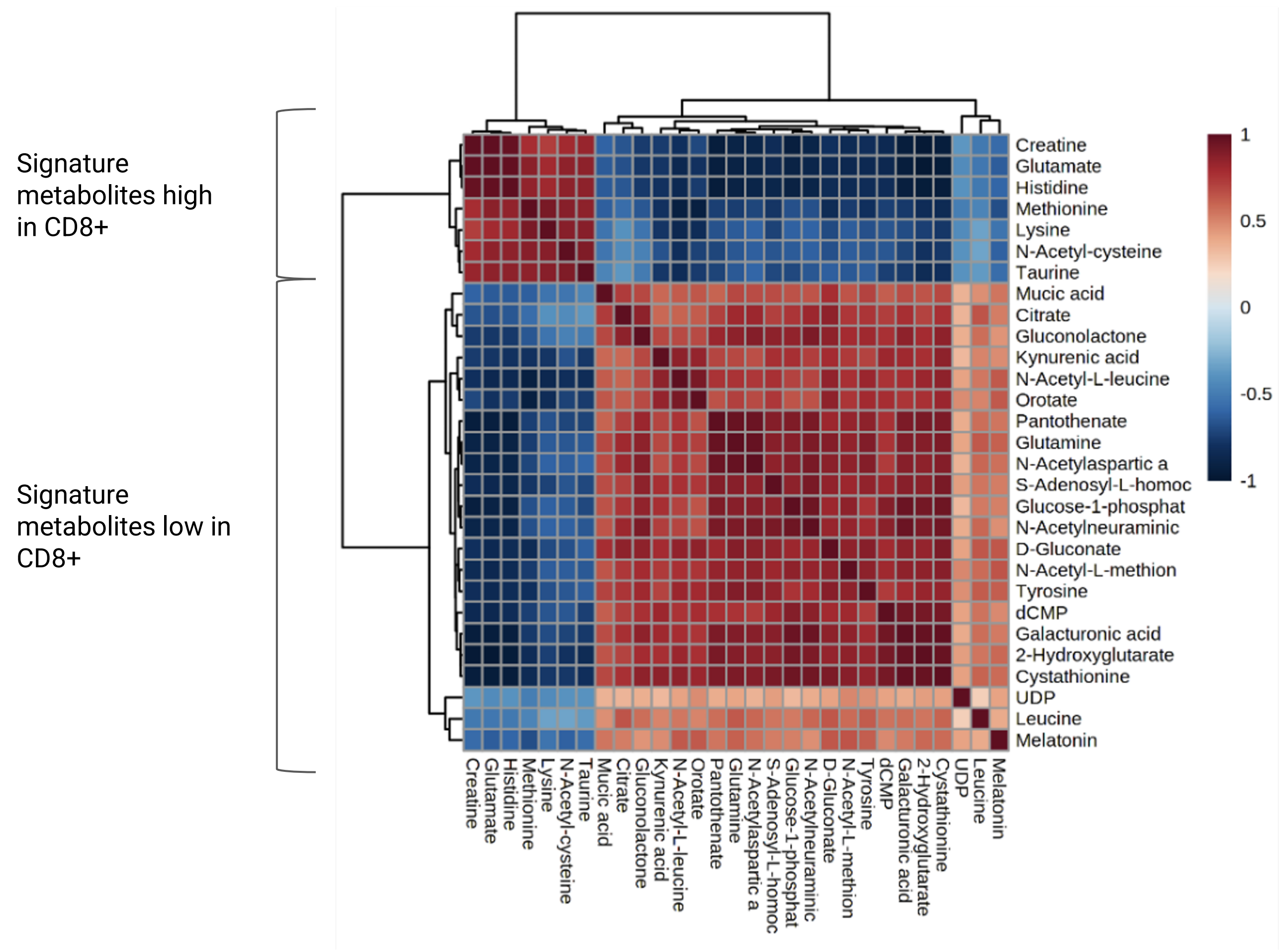

FOXP3

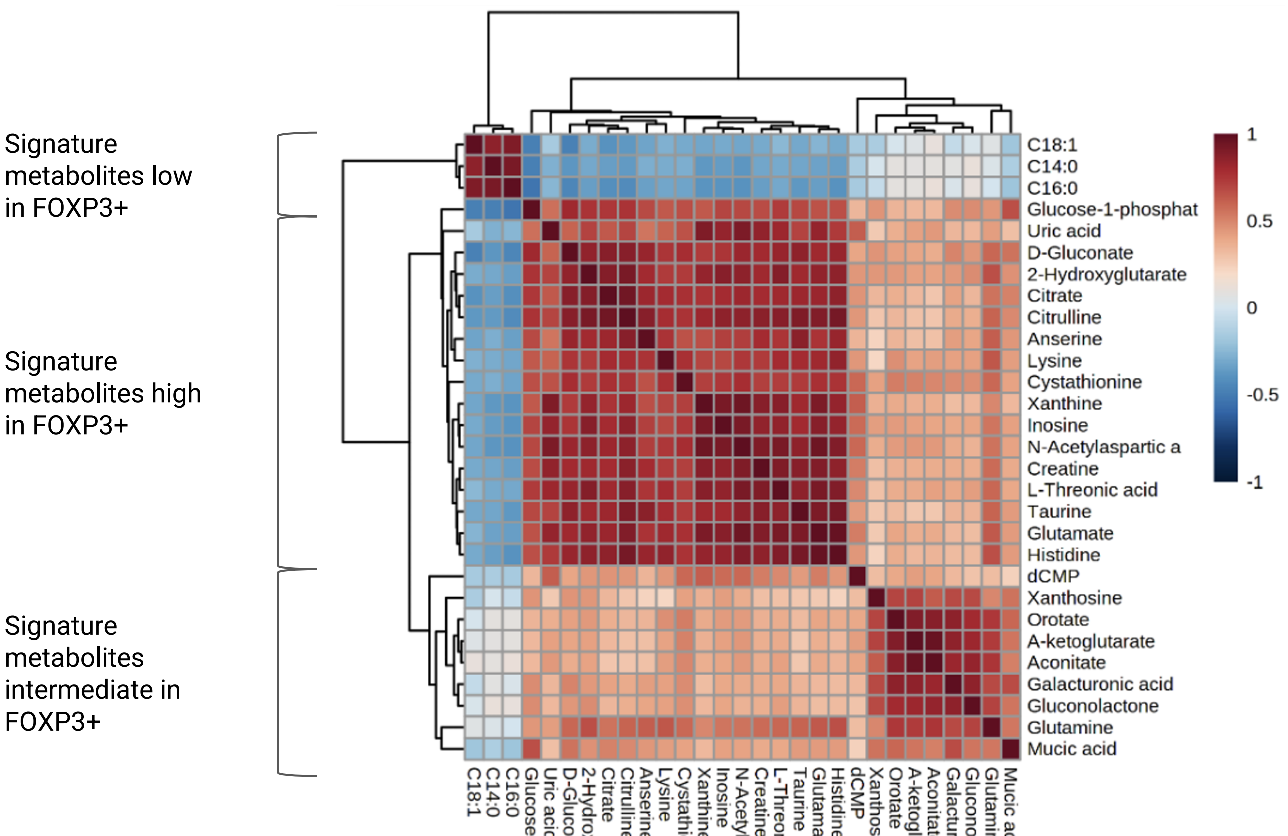

Fig.6

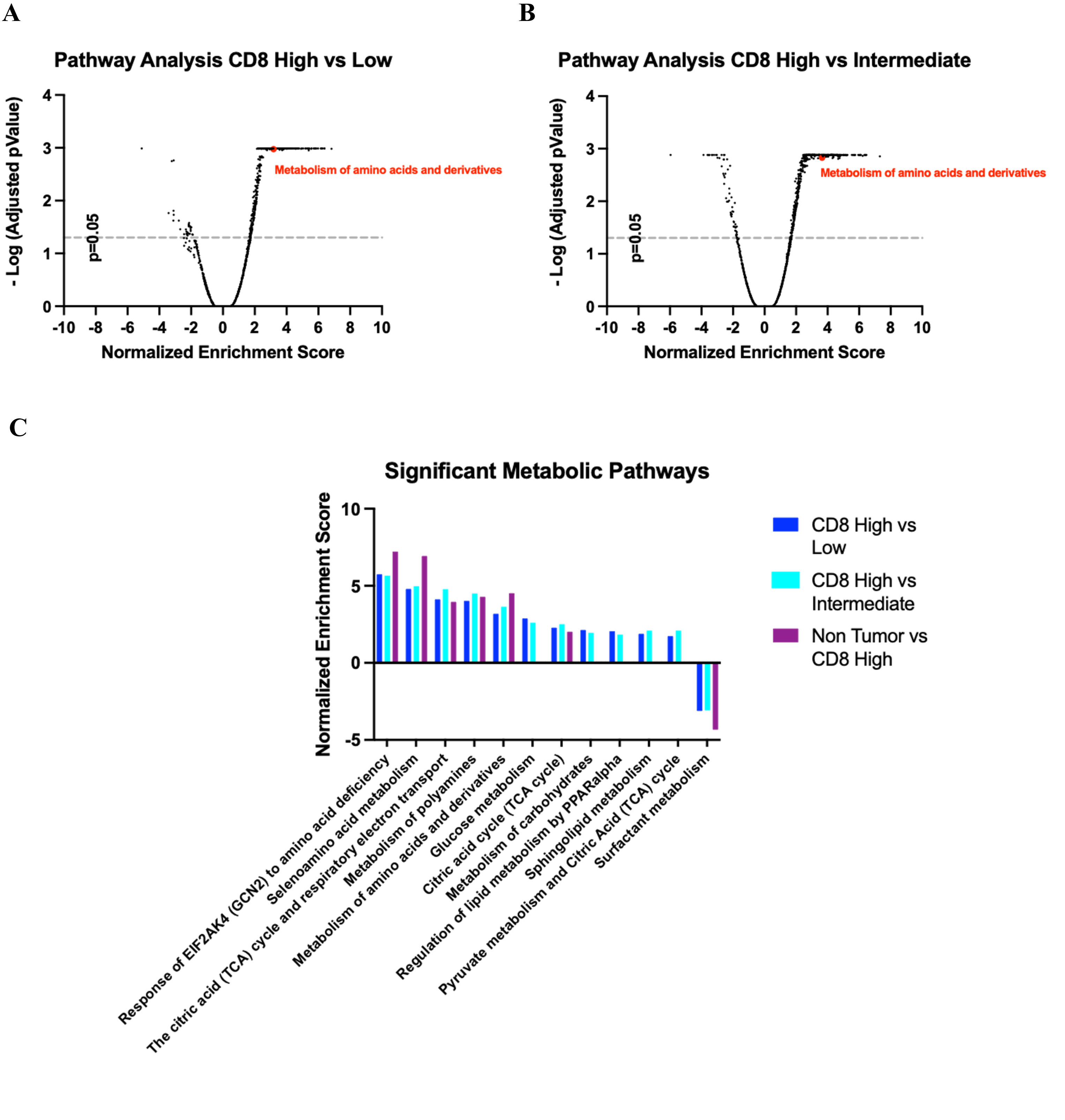
A
Pathway Analysis CD8 High vs Low
- Log (Adjusted pValue)
p=0.05
Metabolism of amino acids and derivatives
Normalized Enrichment Score
B
Pathway Analysis CD8 High vs Intermediate
- Log (Adjusted pValue)
p=0.05
Metabolism of amino acids and derivatives
Normalized Enrichment Score
C
Significant Metabolic Pathways
Normalized Enrichment Score
CD8 High vs Low
CD8 High vs Intermediate
Non Tumor vs CD8 High
Response of EIF2AK4 (GCN2) to amino acid deficiency
Selenoamino acid metabolism
The citric acid (TCA) cycle and respiratory electron transport
Metabolism of polyamines
Metabolism of amino acids and derivatives
Glucose metabolism
Citric acid cycle (TCA cycle)
Metabolism of carbohydrates
Regulation of lipid metabolism by PPARalpha
Sphingolipid metabolism
Pyruvate metabolism and Citric Acid (TCA) cycle
Surfactant metabolism

Fig.7

SUPPLEMENTARY FIGURES

A

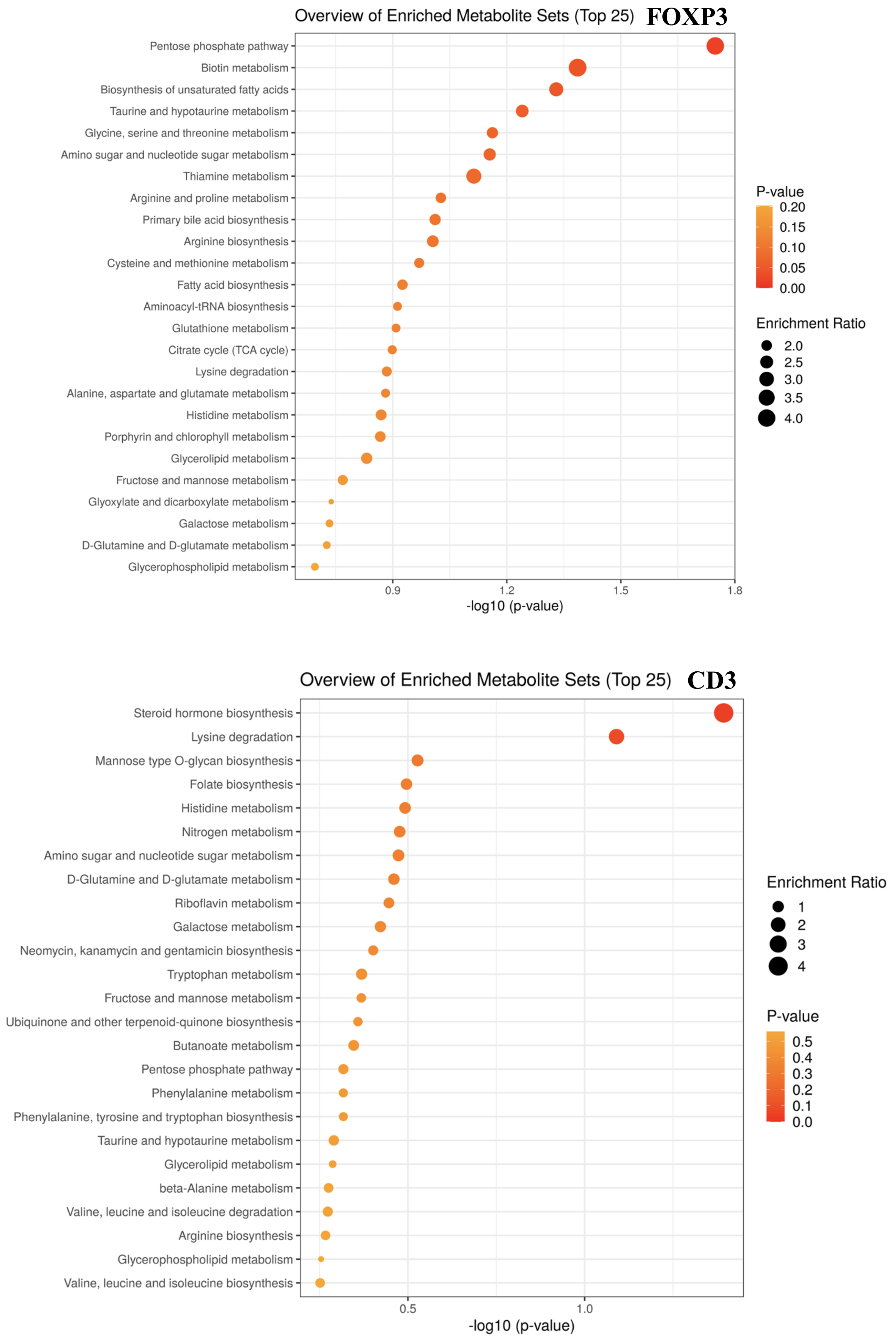

Fig.S1

A) Top 25 most significant pathways enriched comparing FOXP3 high vs FOXP3 low ROIs.

B) Top 25 most significant pathways enriched comparing FOXP3 high vs FOXP3 low ROIs

A

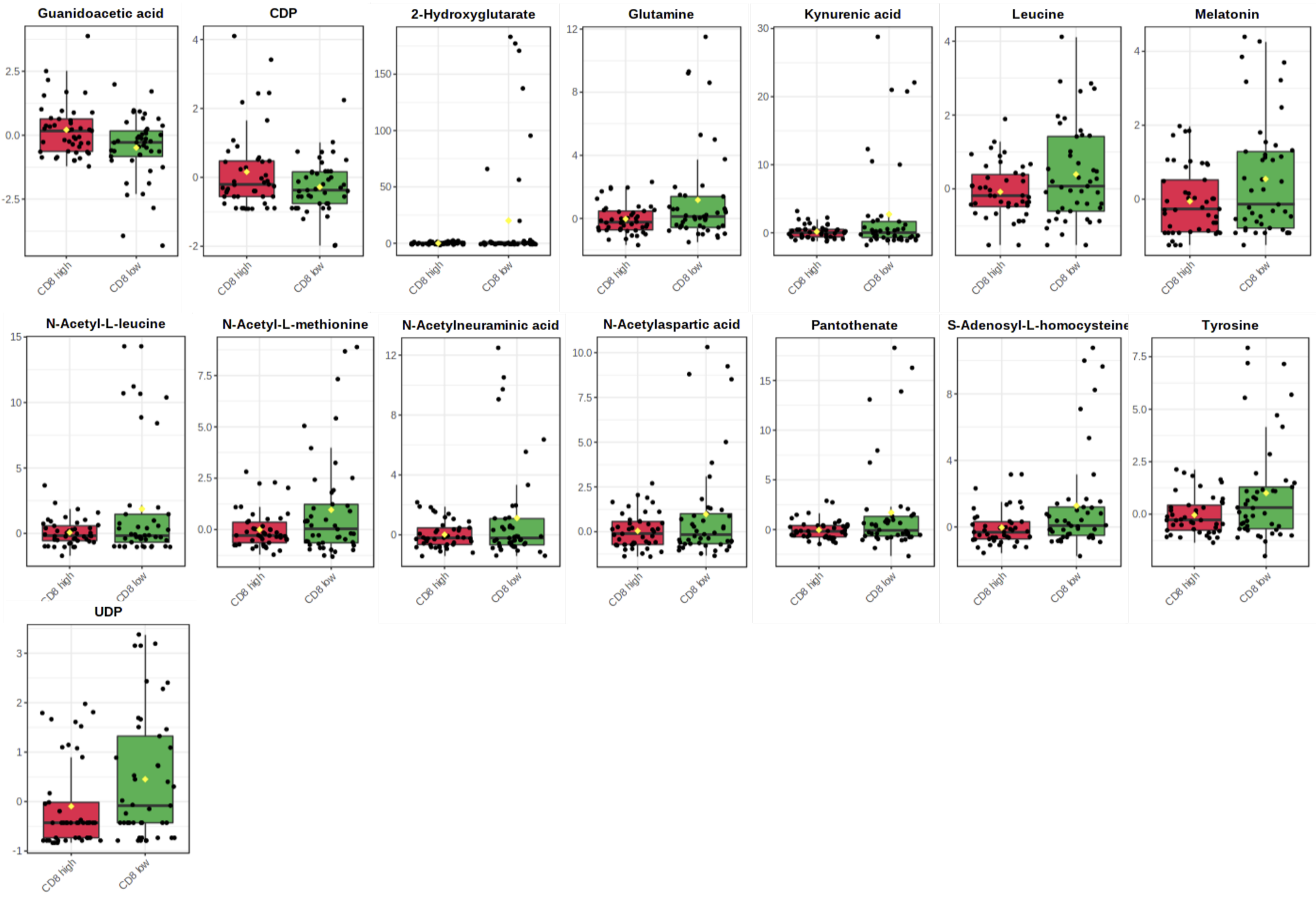

B

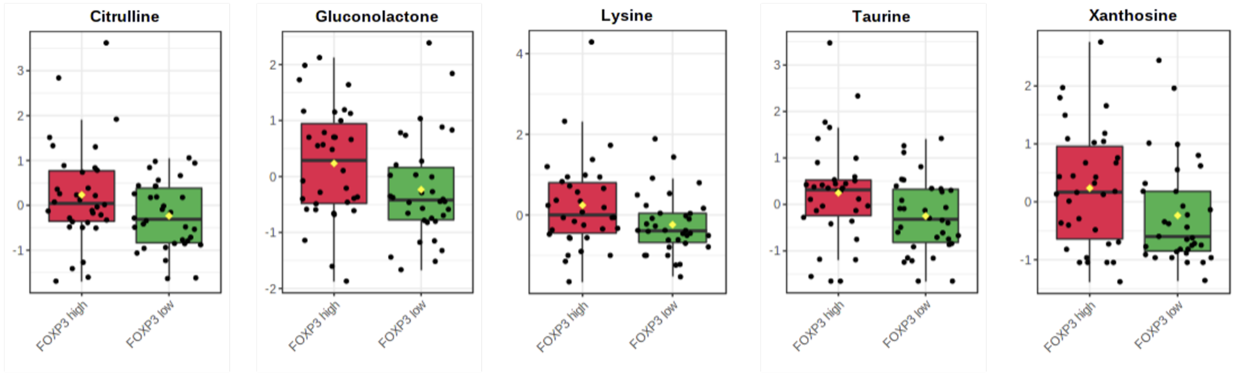

Fig.S2

A) Single metabolite enrichment representation for CD8 enriched ROIs. **B)** Single metabolite enrichment representation for FOXP3 enriched ROIs (significant values have $p < 0.05$).
